# A Robust Protocol for Using Multiplexed Droplet Digital PCR to Quantify Somatic Copy Number Alterations in Clinical Tissue Specimens

**DOI:** 10.1371/journal.pone.0161274

**Published:** 2016-08-18

**Authors:** Curtis B. Hughesman, X. J. David Lu, Kelly Y. P. Liu, Yuqi Zhu, Catherine F. Poh, Charles Haynes

**Affiliations:** 1 Department of Oral Medical Biological Sciences, Faculty of Dentistry, University of British Columbia, Vancouver, British Columbia, V6T 1Z3, Canada; 2 Michael Smith Laboratories, University of British Columbia, Vancouver, British Columbia, V6T 1Z4, Canada; 3 Department of Pathology and Laboratory Medicine, University of British Columbia, Vancouver, British Columbia, V6T 2B5, Canada; 4 Department of Integrative Oncology, British Columbia Cancer Research Centre, Vancouver, British Columbia, V5Z 1L3, Canada; 5 RES’EAU NSERC Research Network, Department of Chemical and Biological Engineering, University of British Columbia, Vancouver, British Columbia, V6T 1Z3, Canada; Tecnologico de Monterrey, MEXICO

## Abstract

The ability of droplet digital PCR (ddPCR) to accurately determine the concentrations of amplifiable targets makes it a promising platform for measuring copy number alterations (CNAs) in genomic biomarkers. However, its application to clinical samples, particularly formalin-fixed paraffin-embedded specimens, will require strategies to reliably determine CNAs in DNA of limited quantity and quality. When applied to cancerous tissue, those methods must also account for global genetic instability and the associated probability that the abundance(s) of one or more chosen reference loci do not represent the average ploidy of cells comprising the specimen. Here we present an experimental design strategy and associated data analysis tool that enables accurate determination of CNAs in a panel of biomarkers using multiplexed ddPCR. The method includes strategies to optimize primer and probes design to cleanly segregate droplets in the data output from reaction wells amplifying multiple independent templates, and to correct for bias from artifacts such as DNA fragmentation. We demonstrate how a panel of reference loci can be used to determine a stable CNA-neutral benchmark. These innovations, when taken together, provide a comprehensive strategy that can be used to reliably detect biomarker CNAs in DNA extracted from either frozen or FFPE tissue biopsies.

## Introduction

Somatic copy number alterations (CNAs) within chromosomes represent a unique class of genetic events shown to correlate with development and progression of cancer [1]. Quantifying CNAs has therefore become fundamental to oncology [2], as evidenced by The Cancer Genome Atlas (TCGA) (http://www.broadinstitute.org/tcga/home), a comprehensive repository of CNAs and other genomic events in major types and subtypes of cancer. The TCGA has revealed, for instance, that copy number gains at chromosome 8q24.1, which include within the myelocytomatosis (*MYC*) oncogene, occur in 45.9% of all cancers. Among its many uses, CNA analysis is finding clinical acceptance and use in cancer diagnostics and theranostics [3]. For example, breast cancer patients positive for gains in the human epidermal growth factor receptor 2 (*HER2)* gene are eligible for treatment with trastuzumab [4], while gains in 14q32.33 are predictive of resistance and progression-free response to platinum therapy in epithelial ovarian cancer [5]. Established methods for quantifying a CNA at genomic loci include fluorescence *in-situ* hybridization (FISH) [6], multiplex ligation-dependent probe amplification (MLPA) [7] and various modalities of quantitative PCR (qPCR) [8]. Several technologies are available for genome-wide analysis of CNAs, including comparative genomic hybridization (CGH) arrays [9, 10], single nucleotide polymorphism (SNP) genotyping arrays [11, 12], and whole-genome next generation sequencing (WG-NGS) [13]. Although these more comprehensive techniques have been used to greatly improve our understanding of CNAs in cancer, they have not gained widespread use in clinical testing, possibly due to challenges imposed by the throughput, cost, sensitivity (*i*.*e*. inability to detect subtle CNAs), and reference material requirements of these methods [14, 15].

Digital PCR [16], most notably droplet digital PCR (ddPCR) [17], is an emerging technology that has been used to quantify CNAs within a genomic target (biomarker) [18], including gains in *HER2* prognostic of breast cancer [19, 20] and in the fibroblast growth factor receptor 2 (*FGFR2*) associated with gastrointestinal tumors [18]. While it cannot provide the comprehensive genomic coverage offered by CGH arrays, SNP arrays or WG-NGS, ddPCR can measure individual CNAs, or in principal panels of CNAs, at a cost and sensitivity appropriate for routine use in clinical settings [21]. To date it has been used to detect alterations in a single biomarker in DNA recovered from either fresh (e.g., blood) or frozen tissues [22–26].

However, clinical testing is very often performed on formalin-fixed paraffin-embedded (FFPE) biopsy specimens. Genomic DNA (gDNA) recovered from FFPE samples is known to suffer from both irreversible and reversible damage that can lower both the quantity and quality of amplifiable material available for testing [27, 28]. Relatively little attention has been given to application of ddPCR to the determination of CNAs in gDNA recovered from FFPE specimens, with the few studies reported limited to quantification of high-level copy number gains in a *single* target (e.g., *HER2* or *FGFR2*) [18, 19, 29]. However, the analysis of multiple genetic markers, often in combination with standard histo-pathologic metrics, is known to greatly improve prediction of risk and progression of cancers [30, 31]. The limited quantity of gDNA typically recovered from clinical FFPE specimens then mandates that ddPCR assays interrogating a panel of biomarkers be multiplexed so as to keep costs in check while maximizing clinically relevant information. Methods for designing multiplexed ddPCR reactions comprised of two or more unique targets per reaction well, and for analyzing the resulting complex data sets, must be developed and proven applicable to gDNA obtained from clinical samples. This latter point is particularly important, as fragmentation and chemical modification of gDNA within FFPE samples can greatly reduce the quantity of amplifiable material. For a panel of target templates of different lengths, fragmentation may bias CNA calls, while irreversible damage in the form of sequence alterations may serve to reduce the amplification efficiency, creating “rain”–defined as droplets with signal lying along a vector connecting two clusters in the ddPCR output [32].

Finally, the reliable use of copy numbers as indicators of disease requires not only the identification of biomarkers whose copy number gains or losses are truly prognostic of disease, but also the identification of one or more loci that can serve as an effective reference for normalizing those CNAs (*i*.*e*., control markers that are CNA neutral relative to the average ploidy of the sample). Biomarkers in ddPCR assays may include loci within exons of specific oncogenes or tumor suppressor genes. However, these two types of biomarkers are susceptible to somatic point mutations (SPMs) within primer or probe hybridization regions that can serve to inhibit amplification or probe hybridization/ hydrolysis, respectively, resulting in false CNA calls [33]. Alternatively, one might target introns or intergenic regions, as prognostic CNAs can occur in chromosomal regions not mapped to specific genes [34]. Finally, microsatellites might be targeted to exploit the fact that LOH data are available to both validate ddPCR assay results and improve diagnostic calls [30, 35].

Presently therefore, the accurate quantification of CNAs by ddPCR remains a formidable challenge due to limitations in current data analysis algorithms and to poorly understood elements of the combined sample-processing method and ddPCR experiment that, together, can serve to bias results. Improved data analysis tools and understanding of experimental artifacts that can skew multiplexed ddPCR data, particularly for FFPE specimens, are required to enable confident identification of subtle genomic changes prognostic of disease and ensure appropriate clinical action is taken. Current algorithms for computing CNAs from ddPCR data sets include those developed by Dube *et al*. [22], by Whale *et al*. [26], and by Dorazio and Hunter [36]. Each of these tools can estimate CNAs within a single biomarker relative to copies of a single reference loci assumed to be CNA neutral during disease progression. Those algorithms are therefore not intended for application to the analysis of multiplexed ddPCR data sets in which a panel of biomarkers is tested both within one reaction well and across multiple reaction wells. Moreover, accurate CNA analysis using any one of these tools is challenged by the required assumption that the chosen reference remains truly CNA neutral during disease progression. That may be true in many cases, but it is not generally true in cancers [37], creating the potential for misleading or uninformative clinical results.

We therefore present here a series of novel strategies and tools for generating and analyzing data from multiplexed ddPCR experiments designed to measure CNAs in a chosen set of biomarkers. These advances include strategies for optimal primer and probe design and a method to achieve non-orthogonal positioning of droplet clusters within the output of multiplexed ddPCR experiments to avoid “rain” from secondary and higher-order droplet clusters overlapping primary droplet clusters and thereby confounding data analyses. We demonstrate how multiple reference loci templates may be selected from a larger panel and used to create a CNA-neutral benchmark that avoids copy number variations in individual reference loci templates that can skew CNA analysis. We also present methods that can be used to design and conduct multiplexed ddPCR experiments to avoid other artifacts and systemic errors that can bias determination of CNAs, particularly for gDNA from FFPE specimens. Specifically, fragmentation is unavoidable from these samples, and we show that loss of biomarker and reference loci due to gDNA fragmentation is template-length dependent; we then provide a method to account for those length-dependent losses when computing CNAs from ddPCR data.

Lastly, we extend the model of Whale *et al*. [26] to include our multi-reference loci benchmark and thereby enable accurate quantification of CNAs for multiple biomarkers using ddPCR assays in which reactions are 4-plexed per well and multiple reaction wells are employed. The performance of the resulting strategy is demonstrated by using multiplexed ddPCR to quantify CNAs in 15 biomarkers within gDNA from frozen tissues representing normal, dysplasia and squamous cell carcinoma (SCC). These samples have previously been studied by CGH array [38], permitting agreement with that orthogonal technique to be used to verify our new method. We also demonstrate that the method is applicable on FFPE samples by analyzing gDNA from sets of FFPE tissues that are field-adjacent to paired specimens collected from the same surgery and then frozen.

## Materials and Methods

### Primers and probes

Primers and probes for each biomarker or reference template were synthesized by IDT Inc. (Coralville, IA). All primers were purified by desalting, while DNA or LNA-bearing dual hydrolysis probes containing either a 5’-FAM or 5’-HEX reporter dye were HPLC purified. All oligonucleotides were resuspended in IDTE buffer (10 mM Tris, pH 8.0, 0.1 mM EDTA) at a total strand concentration *C*_*t*_ = 100 μM and stored at −20°C prior to use. Sequences of all primers and probes used are provided in S1 Table of the Supplementary Data.

### DNA extraction from blood, frozen and FFPE tissue

Blood and oral tissue specimens were collected with written consent from participants, with the consent, collection and de-identification protocols used approved by the UBC Clinical Ethics Research Board (CREB number H09-01255). Blood specimens were collected by drawing 4 ml whole blood into a K_2_-EDTA vacutainer collection tube (BD Diagnostics, NJ, USA). Centrifugation was then used to separate whole blood into plasma and buffy coat fractions stored at -80°C until use. For this study, gDNA was extracted from frozen buffy coat using a QIAamp DNA Blood Mini Kit (QIAGEN Inc. Hilden, Germany) according to the manufacturer’s protocol (QIAamp^®^ DNA Mini and Blood Mini Handbook, 4^th^ Edition, available online at www.qiagen.com/ca/resources/ (May 30, 2016)).

Extraction of gDNA from frozen-tissue biopsies followed protocols previously described [39] for microdissection of 10-μm thick sections. Microdissected tissue was digested and subjected to gDNA extraction using an AllPrep^®^ DNA/RNA/miRNA Universal Kit (QIAGEN) following the manufacturer’s protocol (AllPrep^®^ DNA/RNA/ miRNA Universal Handbook, available online at www.qiagen.com/ca/resources/ (May 30, 2016)).

FFPE biopsy samples were microdissected manually from 10-μm thick, methyl-green stained sections in order to isolate the desired tissue representing the matched histological stage. Microdissected samples were digested by incubation in lysis buffer and proteinase K (PK) at 56°C for 48 hours, with addition of 10-μl fresh PK buffer if there was any undigested material. gDNA was extracted using the QIAamp DNA FFPE Tissue Kit (QIAGEN) according to manufacturer’s protocol (available online (May 30, 2016) at www.qiagen.com/ca/resources/).

### ddPCR workflow

Preparation of 20 μL ddPCR reactions used 10 μL of 2X ddPCR SuperMix for probes (No dUTP) (Bio-Rad Inc., Hercules, CA), 5–20 ng of gDNA quantified by the Qubit dsDNA high sensitivity assay kit (Thermo Fisher Scientific, Waltham, MA), forward primers (FP) and reverse primers (RP), each at a final *C*_*t*_ = 900 nM, and FAM and/or HEX-labeled probes (*C*_*t*_ = 200–600 nM). Droplets were then generated in the QX200 droplet generator (Bio-Rad) by loading 20 μL of the reaction mixture and 70 μL of droplet generation oil for probes (Bio-Rad) onto matched wells of a DG8 cartridge (Bio-Rad). Approximately 45 μL of the droplet/oil mixture (12,000–20,000 droplets) were transferred with an L8-50XLS+ multichannel pipette (Mettler Toledo, Columbus, OH) to a semi-skirted 96-well plate (Bio-Rad). The plate was sealed with a pierce-able foil heat seal using a PX1 PCR plate sealer (Bio-Rad). The plate was loaded and then processed on a PTC-200 thermal cycler (Bio-Rad) using the following amplification protocol: 95°C for 10 min, followed by 50 cycles: denaturation at 94°C for 30 s; annealing at 60°C for 1 min; extension at 65°C for 30 s. Cycling between the temperatures was set to a ramp rate of 2.5°C/sec, and following cycling the sample was held at 98°C for 10 min. Upon completion of the PCR protocol, the plate was read using the QX200 droplet reader (Bio-Rad) with the following settings; channel 1 = FAM and channel 2 = HEX. Droplet counts and amplitudes were then exported to and analyzed with QuantaSoft^™^ software (Bio-Rad).

### CGH array data analysis

For four histologically and geographically distinct sections taken from a surgical field biopsy of an oral squamous cell carcinoma (OSCC) patient, measured CGH array log_2_ signal intensity ratios for those probes mapping onto the set of loci used as biomakers in our multiplex ddPCR experiments were taken from Tsui *et al*. [38]. CGH array probes overlaying and/or proximal to each biomarker were identified using the UCSC Genome Browser NCBI35/hg17 assembly [40], and the resulting set of log_2_ values for the probes were then averaged for comparison to CNAs for the corresponding biomarker(s) in that locus assayed by multiplexed ddPCR.

## Results and Discussion

### Primer and Probe Designs Can Serve to Reduce Rain

**Fig 1:**
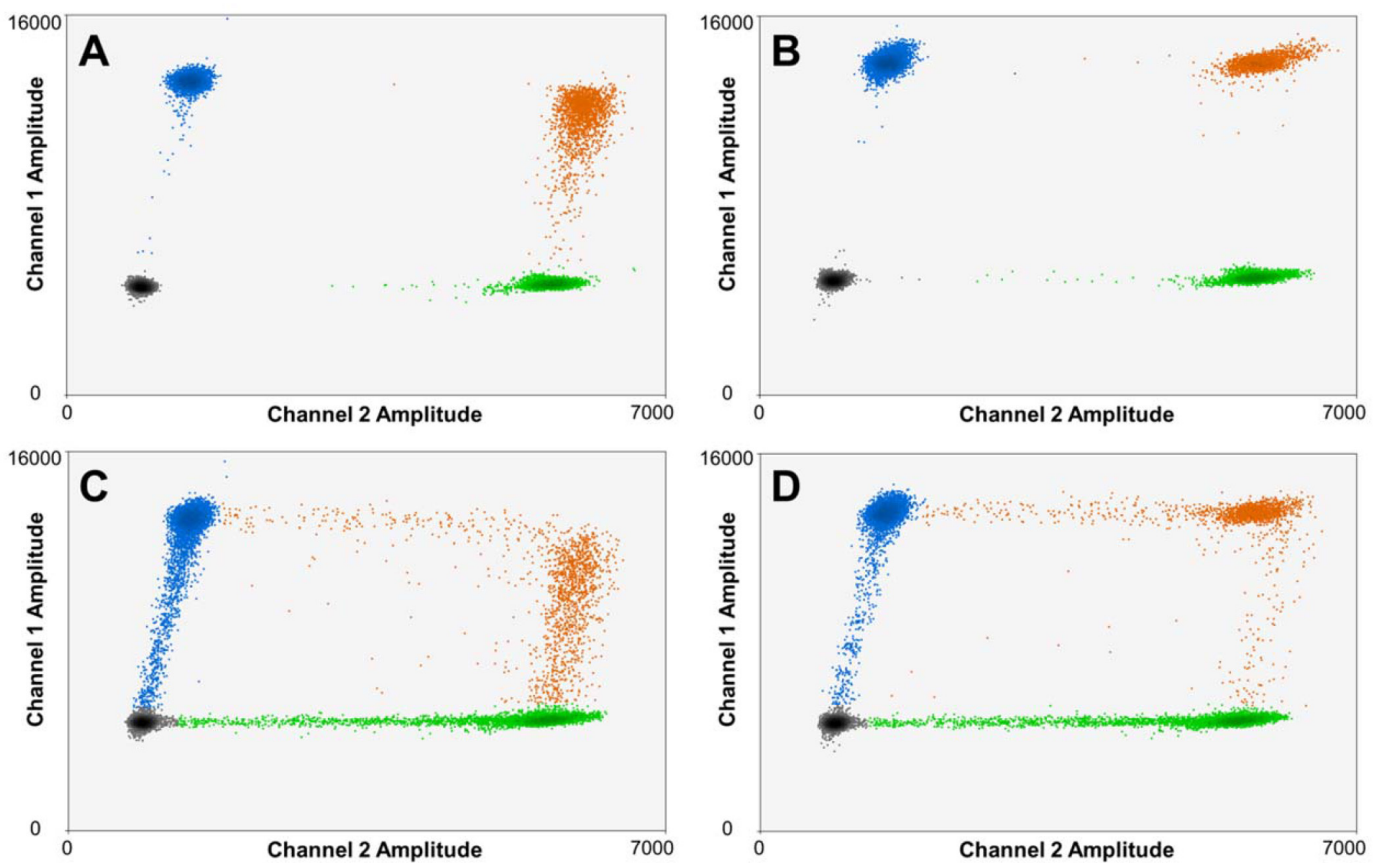
Overlayed output data for a set of *n* = 4 duplex ddPCR experiments amplifying the *CPT2* (HEX) reference on 1p32 and the *D4S1652* (FAM) micro-satellite biomarker, a ATCT tetranucleotide repeat found on 4q35. (**A**) gDNA from normal frozen blood amplified using primer/probe sets designed using standard Primer 3 software. A disperse secondary cluster and dense rain between clusters are observed. (**B**) Amplification of the same sample using primer/probe sets optimized according to the guidelines in S2 Table. More tightly focused clusters and a reduction in rain are achieved. (**C**) gDNA recovered from a normal FFPE sample amplified using the same primer/probe sets as in (A). Significant levels of rain and poorly focused clusters are observed. (**D**) gDNA recovered from FFPE tissue amplified using the optimized primer/probe sets. Rain levels are greatly reduced and the clusters are tightly focused.

Rain is an innate feature in the output of ddPCR experiments [32]. For a standard single-plex experiment, ddPCR output data for DNA extracted from fresh tissue, including normal blood, often exhibits tightly focused droplet clusters and a low level of rain that tends to be relatively insensitive to primer and probe designs (S1 Fig). More significant levels of rain and less focused clusters are generally observed when reactions are duplexed or multiplexed to higher degrees within a well of a ddPCR experiment (Fig 1A). Optimization of the multiple probe and primer sets is then required to achieve tightly focused primary and secondary droplet clusters and minimal rain (Fig 1B). That optimization process can be facilitated by knowledge of sources of rain, which have not been fully described for multiplexed ddPCR experiments. In our multiplexed studies on gDNA isolated from normal blood, we observe three distinct types of rain that can be classified as primary rain, secondary rain, and late rain, respectively. S2 Table provides a description of each type of rain and its formation mechanism(s), as well as primer and probe design strategies that may be employed to mitigate its occurrence in multiplexed ddPCR experiments on DNA extracted from blood or from frozen tissues (also see S1 to S3 Figs for examples of each type of rain).

Rain is generally far more prevalent in ddPCR data for DNA containing a heterogeneous pool of templates [32], including that obtained from clinical FFPE specimens (Fig 1C). Irreversible sequence alterations are often observed in DNA extracted from FFPE tissue, with C>T and G>A transitions being the most prevalent [28]. Sequence alterations in the probe recognition site of a template can reduce the efficiency of probe hybridization and hydrolysis, resulting in lower end-point fluorescence signals for droplets containing those damaged templates. Sequence artifacts can also affect the performance of primers, especially if the damage to the template occurs at or near the sequence that hybridizes to the 3’ end of the primer or if fragmentation shortens the primer hybridization site on the template. Template amplification efficiency may thereby be reduced to produce “rain” droplets.

Though optimization of probe and primer designs is generally required to reduce rain and focus droplet clusters within ddPCR output for FFPE specimens, spare clinical material that might be used for that purpose is often not available. We have found, however, that DNA extracted from more readily available normal blood samples may be used in combination with the design strategies defined in S2 Table to optimize probe and primer sets for a multiplexed ddPCR experiment applied to gDNA from FFPE samples. This is demonstrated in Fig 1D, where a significant reduction in the density of rain is achieved in a duplex ddPCR experiment on the same FFPE specimen as before (i.e., Fig 1C) by using probe and primers sets optimized on normal blood (Fig 1B).

This optimization is generally required, as we find that primer or probe inefficiencies observed in multiplexed reactions can bias copy number ratios. Take for example the four data sets reported in Fig 1 for a duplex reaction targeting the *CPT2* gene within chromosome 1p32, which is taken as a reference (*r*) locus here, and the *D4S1652* microsatellite locus, a highly conserved ATCT tetranucleotide repeat sequence found on 4q35, which is taken as the informative biomarker (*i*). In normal human tissue, a copy number ratio *R*_*i/r*_ (*i* = *D4S1652*, *r* = *CPT2*) of unity is expected for these two markers. Whale *et al*.[26] have shown *R*_*i/r*_ can be computed from the *CPD* of each template (*t*)

$$CPD_{t}=-\text{ln}\left(1-\frac{p_{t}}{C}\right)$$ (1)

where *p*_*t*_ is the total number of droplets positive for template *t* (*i* or *r*) and *C* is the total droplet count. *R*_*i/r*_ for the two templates, along with the associated standard deviation $\sigma_{R_{i/r}}$, is then given by

$$\text{ln}\left(R_{i/r}\right)=\text{ln}\left(\frac{CPD_{i}•C}{CPD_{r}•C}\right)=\text{ln}\left(\frac{CPD_{i}}{CPD_{r}}\right)$$ (2)

and

$$\text{ln}\left(\sigma_{R_{i/r}}\right)\approx \sqrt{\left(\frac{1-e^{-CPD_{i}}}{C{\left(CPD_{i}\right)}^{2}e^{-CPD_{i}}}\right)+\left(\frac{1-e^{-CPD_{r}}}{C{\left(CPD_{r}\right)}^{2}e^{-CPD_{r}}}\right)}$$ (3)

Table 1 reports the *R*_*i/r*_, along with the high and low 95% confidence intervals, for each ddPCR dataset shown in Fig 1. For the blood sample, an *R*_*i/r*_ of unity is indeed recorded (within experimental error) using either primers (S1 Table) for this biomarker designed using conventional tools (Fig 1A), or primers optimized using the strategies defined in S2 Table (see also Fig 1B). However, when the standard primers are then applied to DNA isolated from a normal FFPE sample, a high degree of primary-type rain is observed (Fig 1C), particularly for the primary cluster of droplets containing the biomarker *D4S1652* –indicative of inefficient amplification. An anomalously low *CPD*_*i*_ is thereby recorded, resulting in a bias in the *R*_*i/r*_ value computed from it (Table 1). Optimization of the primer set for the *D4S1652* marker greatly reduces primary rain (Fig 1D) and eliminates the bias in *R*_*i/r*_ (Table 1).

**Table 1:** Primer/probe sets must be optimized to produce unbiased high-quality data from multiplexed ddPCR experiments. *R*_*i/r*_ (*i* = *D4S1652*, *r* = *CPT2*) values and low and high 95% confidence intervals (CIs) computed from each ddPCR dataset shown in Fig 1.

Specimen	Primer/ Probe set	CPD		*R*_*i/r*_		
		*D4S1652*	*CPT2*	Mean	95% CI	
					Low	High
Normal Blood	Standard	0.220	0.221	1.00	0.96	1.03
	Optimized	0.225	0.226	1.00	0.96	1.03
Normal FFPE tissue	Standard	0.185	0.201	0.92	0.89	0.95
	Optimized	0.196	0.194	1.01	0.98	1.04

### Clusters Can Be Positioned Non-Orthogonally to Avoid Interference from Rain

**Fig 2:**
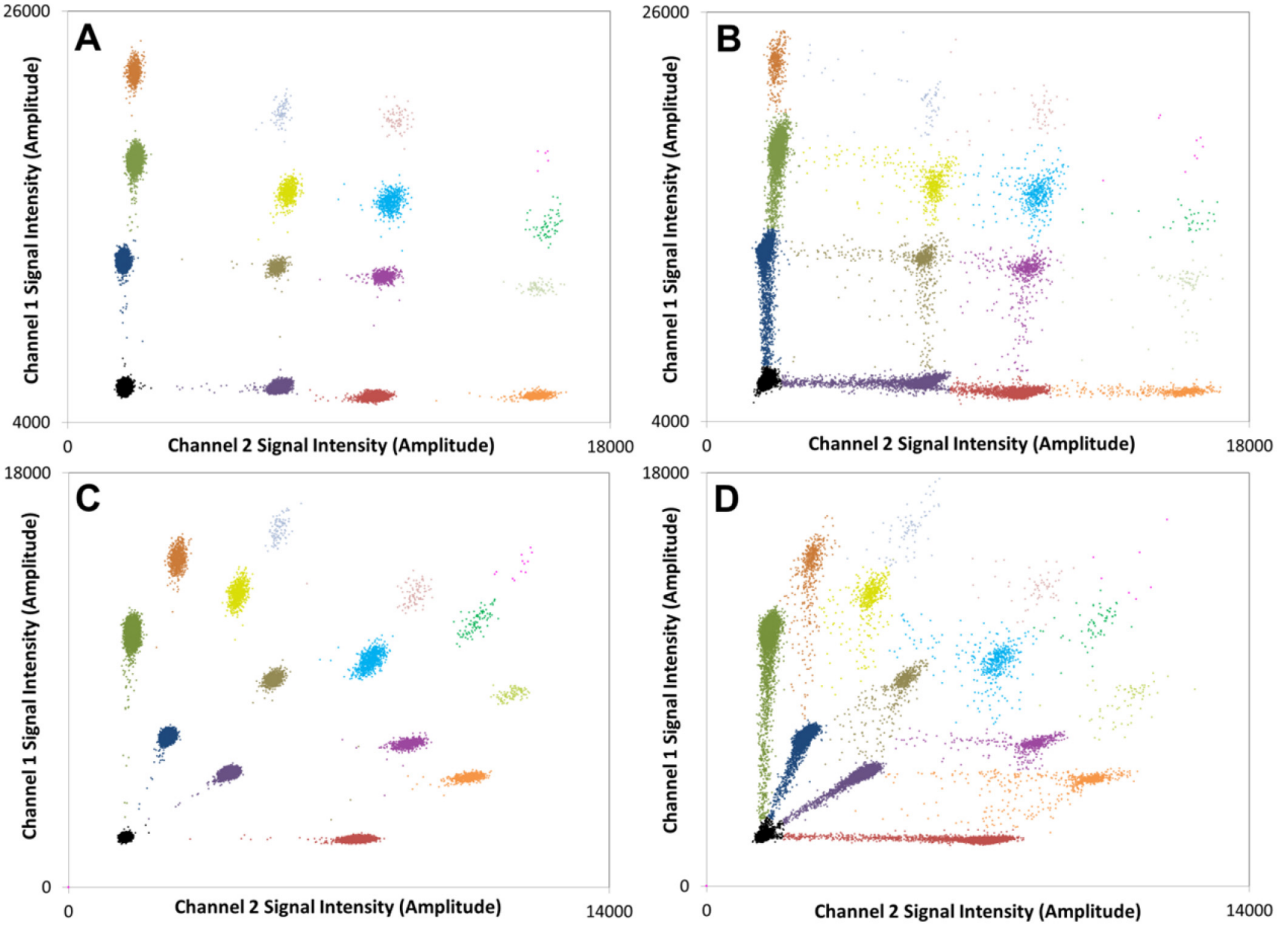
Multiplex (4-plex) ddPCR output generated using either a standard orthogonal layout of droplet clusters or our staggered layout technique. Standard orthogonal layout of droplet data for DNA extracted from (**A**) normal blood or (**B**) normal FFPE tissue. Staggered layout of droplet data for DNA extracted from (**C**) normal blood or (**D**) normal FFPE tissue. The droplets shown in each plot are a combination of *n* = 4 replicates and the four primary clusters are labelled. The four loci amplified were 1 = *ACADM*; 2 = *KCNS3*; 3 = *SLC25A12* and 4 = *HFE2*. The orthogonal layout was achieved using the following concentrations of labelled probes (1 = 600 nM FAM; 2 = 300 nM FAM; 3 = 300 nM HEX; 4 = 600 nM HEX). The staggered layout was achieved using the recipe described in main text. Each plot was created by the overlayed output data for a set of *n* = 4 multiplex ddPCR experiments.

The BioRad QX100 or QX200 ddPCR reader offers only two unique channels for monitoring fluorescence emission intensities (typically from FAM and HEX labeled probes, respectively). Triplex and higher order multiplexing of template amplification reactions per well requires a means to segregate and assign within this two-channel readout the larger diversity of unique template-positive droplet clusters formed. Take, for example, a 4-plexed reaction in which amplification of two of the targets is monitored with sequence-specific FAM-labeled probes, and that of the other two targets with sequence-specific HEX-labeled probes. One reported strategy [41] for segregating the droplets according to the template(s) they each contain is to use different concentrations of each FAM-labeled probe, and likewise of each HEX (or VIC) labeled probe, such that droplet clusters in the ddPCR output align orthogonally. Fig 2A demonstrates the output of this approach for a 4-plex ddPCR experiment on gDNA obtained from fresh blood. Predictable and well-defined positioning of primary and higher-order droplet clusters is achieved.

However, limitations to orthogonal layout of droplet clusters become apparent when the method is applied to DNA from FFPE material (Fig 2B). Rain from clusters of higher channel 1 or 2 amplitude overlaps clusters (and their associated rain) of lower channel 1 or 2 signal intensity, respectively. This makes it difficult to de-convolute the ddPCR output in a manner that allows reliable assignment of each droplet. A tendency to over-assign droplets to lower-signal-intensity clusters is generally created, which can result in systemic errors in CNAs computed from the cluster assignments. For example, *R*_*i/r*_ values computed from the data in Fig 2B are both *ca*. 1.2 (Table 2), despite the fact that the targets, which in this example are all reference loci, are expected to be present at the same abundance within the FFPE sample.

**Table 2:** Staggered layout of droplet clusters eliminates interference from rain that can bias CNA values for gDNA from clinical specimens. *R*_*i/r*_ values and low and high 95% confidence intervals computed from each ddPCR dataset shown in Fig 2.

Specimen	*i/r*	Orthogonal Layout			Staggered Layout		
		*R*_*i/r*_	95% C.I.		*R*_*i/r*_	95% CI	
			Low	High		Low	High
Normal Blood	*KCNS3/ACADM*	0.97	0.93	1.00	1.04	1.01	1.08
	*SLC25A12/HFE2*	1.04	1.00	1.07	1.02	0.99	1.05
Normal FFPE tissue	*KCNS3/ACADM*	1.21	1.17	1.26	1.03	0.99	1.06
	*SLC25A12/HFE2*	1.21	1.16	1.25	1.03	1.00	1.07

We therefore sought ways to eliminate this bias through creation of an alternative strategy in which primary and higher order clusters are made to adopt a non-orthogonal layout, as demonstrated in Fig 2C for a normal blood sample. In that 4-plex reaction, we achieve staggered segregation of all positive droplet clusters by labeling the probe against target 1 with FAM (300 nM probe total), a portion of the probe against target 2 with FAM (140 nM) and the remainder with HEX (60 nM), a portion of the probe against target 3 with HEX (140 nM) and the remainder with FAM (60 nM), and all of the probe against target 4 with HEX (300 nM). Staggered patterning of droplet clusters can therefore be achieved by 1) varying the concentration of the different probes used, and 2) creating two different labeled forms of a subset of those probes. These two levers can be manipulated (the concentrations and labeling ratios used in the example above are just illustrative of the approach) to tune the positions and spacing of positive droplet clusters to allow unambiguous droplet assignments.

The benefit of this approach is seen especially when gDNA from FFPE material is analyzed in a 4-plex reaction. In the staggered layout (Fig 2D), rain from clusters of higher signal intensity does not interfere with clusters of lower signal intensity, and a significant reduction in signal overlap is thereby achieved; we note that rain trails from secondary and higher-order clusters do cross, but the uncertainty in droplet assignments is nevertheless greatly reduced. As a result, *R*_*i/r*_ values computed from the non-orthogonal data set (Fig 2D) show no bias (Table 2).

### Quantifying and Accounting for Fragmentation of Template DNA

DNA extracted from FFPE material is known to be highly fragmented, often comprised of segments that are, on average, several hundred base pairs in length [42]. Highly fragmented gDNA can have significantly lower read coverage in NGS [43]. Fragmentation of gDNA can likewise influence ddPCR output [44], though its precise effect on ddPCR-based analysis of CNAs is not well understood. Shear-induced fragmentation of gDNA is known to occur stochastically and at a frequency that correlates with length [27, 45]. Thus, in duplexed ddPCR experiments designed to detect a CNA (i.e. an *R*_*i/r*_) in a prognostic biomarker *i* relative to a single co-amplified reference locus *r*, any bias created from DNA fragmentation can in principal be mitigated by forcing the two templates to be of similar length [28]. But implementation of that solution becomes more problematic and restrictive in more highly multiplexed assays. For example, direct comparison of CNAs determined by ddPCR with those obtained by other techniques used to measure allelic imbalances, such as LOH assays, may require use of templates of varying lengths. We therefore sought to determine if any systemic errors occur in CNAs determined by ddPCR due to DNA fragmentation and, if so, how those errors can be corrected to yield the true CNA for each biomarker analyzed.

**Fig 3:**
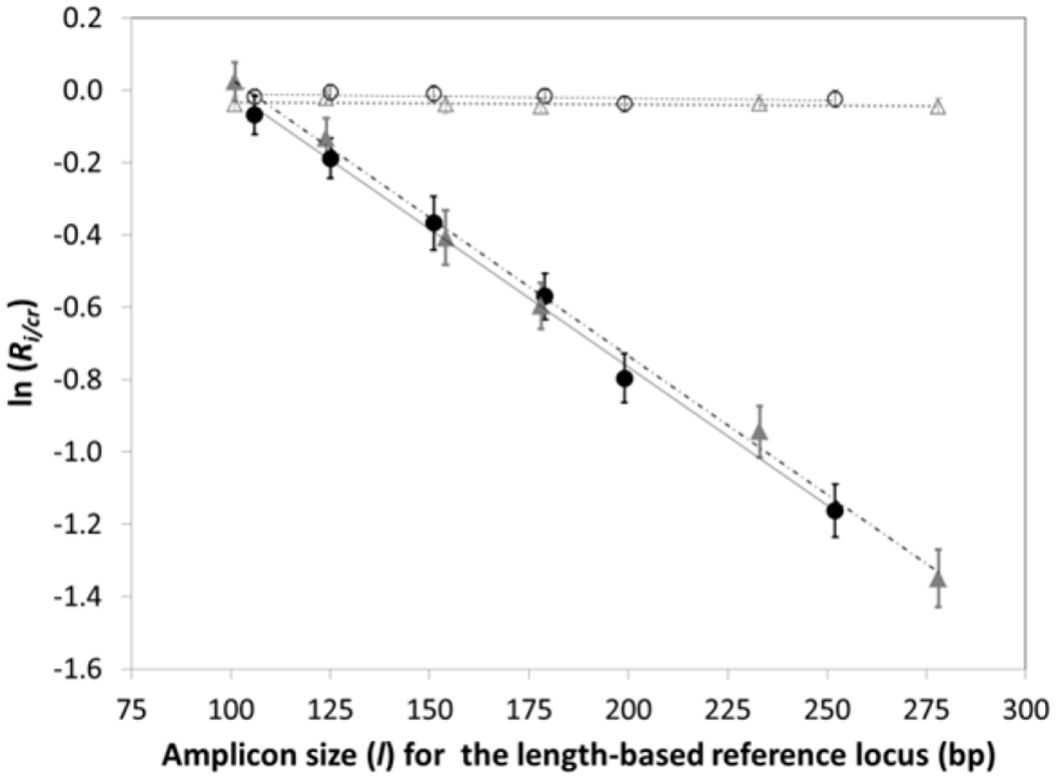
Analysis of DNA fragmentation as a function of template length and type of sample. ln (*R*_*i/cr*_)_*M*_ values determined from triplex ddPCR experiments on gDNA recovered from normal blood (open symbols) or FFPE tissue specimens (filled symbols). Each triplex experiment co-amplifies the *HER2 cr* and one length *i* each of the *CPT2* length-based control (circles) and the *KCNS3* length-based control (triangles). Error bars represent a 95% confidence interval.

The ability of digital PCR to accurately quantify concentrations of amplifiable template provides a potential method to evaluate differences in the frequency of fragmentation of templates of different length and their impact on CNAs computed from ddPCR data. We explored this concept by selecting two loci on different chromosomes, *CPT2* at 1p32.3 and *KCNS3* at 2p24.2. For each, we designed a series of *n*_*lc*_ templates of increasing length by varying the location of the FP and/or RP flanking a locus-specific probe hybridization site. We also created a primer/probe set for amplifying a 97 bp template within *HFE2* to serve as a constant length reference (*cr*) for this analysis. Triplex ddPCR experiments co-amplifying the *HER2 cr* and one length *i* of *CPT2* and *KCNS3* were completed to generate *n*_*lc*_ = 6 different *R*_*i/cr*_ values for each length-based template, as reported in Fig 3 for gDNA isolated from either normal blood or FFPE specimens, respectively. The results show that ln(*R*_*i/cr*_) depends linearly on the difference in the length (Δ*l*) of the *CPT2* or *KCNS3* template relative to that of *HFE2*, with a slope that depends on sample type. For DNA isolated from normal blood, the measured *R*_*i/cr*_ (hereafter denoted (*R*_*i/cr*_)_*M*_, where (*R*_*i/cr*_)_*M*_ is computed from the raw ddPCR data using Eqs 1 and 2) for either *CPT2* or *KCNS3* is found to be statistically insensitive to Δ*l*. But for DNA from FFPE tissue, where significant levels of fragmentation are expected, ln(*R*_*i/cr*_)_*M*_ values depend linearly on Δ*l*, creating a systemic error in CNA values that increases with increasing Δ*l*. For *CPT2*, (*R*_*i/cr*_)_*M*_ = 1, as expected for a normal tissue sample, when Δ*l* = 0. However, when Δ*l* = 146, an erroneous (*R*_*i/cr*_)_*M*_ of 0.34 is obtained, illustrating the significant error fragmentation effects can introduce into CNA values computed from ddPCR data for FFPE material.

The strict semi-logarithmic dependence of (*R*_*i/cr*_)_*M*_ on Δ*l* is consistent with the stochastic nature of shear-induced fragmentation events. More importantly, it provides a means to correct (*R*_*i/cr*_)_*M*_ values, where *i* is now any locus of interest, for errors associated with the difference in the length (*l*_*i*_) of *i* relative to that of the constant length reference (*l*_*cr*_). That correction is given by

$$\text{ln}{\left(R_{i/cr}\right)}_{A}=\text{ln}{\left(R_{i/cr}\right)}_{M}+\text{ln}{\left(R_{i/cr}\right)}_{frag}$$ (4)

where

$$\text{ln}{\left(R_{i/cr}\right)}_{frag}=-m_{frag}\left(l_{i}-l_{cr}\right)=-m_{frag}\Delta l$$ (5)

Here, ln(*R*_*i/cr*_)_*frag*_ converts the measured CNA for *i* to its correct or actual (*A*) value, denoted ln(*R*_*i/cr*_)_*A*_, and *m*_*frag*_ is the slope of the ln(*R*_*i/cr*_)_*M*_ versus Δ*l* plot (*e*.*g*., Fig 3). In our method, correcting for errors in CNAs due to differences in template lengths therefore requires the amplification of a constant-length reference template *cr*, as well as a length-based control series, from which the required ln(*R*_*i/cr*_)_*frag*_ value is determined. We note that because ln(*R*_*i/cr*_)_*frag*_ does not depend on the intercept of the ln(*R*_*i/cr*_)_*M*_ versus Δ*l* plot, the correction method does not require the chosen constant reference or length-based control to be CNA-neutral. This makes the choice of loci used as the length-based control and constant-length reference completely arbitrary, as evidenced in part by the identical slopes obtained for the two (*CPT2* (1p32.3) and *KCNS3* (2p24.2)) length-based controls analyzed here.

The standard deviation from the mean (*R*_*i/cr*_)_*A*_ value is given by

$$\text{ln}{\left(\sigma_{R_{i/cr}}\right)}_{A}=\sqrt{\text{ln}{\left(\sigma_{R_{i/cr}}\right)}_{M}{}^{2}+\text{ln}{\left(\sigma_{R_{i/cr}}\right)}_{frag}{}^{2}}$$ (6)

where ln(*σ*_*R*_)_*M*_ is given by Eq 3, and

$$\text{ln}{\left(\sigma_{R_{i/cr}}\right)}_{frag}=\sqrt{\text{ln}{\left(\sigma_{R_{i/cr}}\right)}_{frag,cr}{}^{2}+\text{ln}{\left(\sigma_{R_{i/cr}}\right)}_{frag,lc}{}^{2}}$$ (7)

with

$$\text{ln}{\left(\sigma_{R_{i/cr}}\right)}_{frag,cr}=\sigma_{\text{ln}\left(R_{i/cr}\right),l_{i}}\sqrt{\left(\frac{1}{n_{lc}}\right)+\frac{{\left(l_{cr}-{\bar{l}}_{lc}\right)}^{2}}{\sum_{j=1}^{n}{\left(l_{j}-{\bar{l}}_{lc}\right)}^{2}}}$$ (8A)

$$\text{ln}{\left(\sigma_{R_{i/cr}}\right)}_{frag,lc}=\sigma_{\text{ln}\left(R_{i/cr}\right),l_{i}}\sqrt{\left(\frac{1}{n_{lc}}\right)+\frac{{\left(l_{i}-{\bar{l}}_{lc}\right)}^{2}}{\sum_{j=1}^{n}{\left(l_{j}-{\bar{l}}_{lc}\right)}^{2}}}$$ (8B)

Here $\sigma_{\text{ln}\left(R_{i/cr}\right),l_{i}}$ is the uncertainty in the linear regression of *m*_*frag*_ from ln(*R*_*i/r*_)_*M*_ versus Δ*l* data, ${\bar{l}}_{lc}$ is the average length of the length-based control series, and *j* indexes the *n*_*lc*_ length-based controls used.

### Creating a Stable CNA-Neutral Benchmark for Precise CNA Quantification Relative to the Average Ploidy of the Sample

CNAs are known to occur throughout the genome in all cancer types. Beroukhim *et al*. [37] found that, on average, 33% of the genome of any tumor displays some type of alteration (with gains and losses occurring with roughly equal frequency), while less than 0.5% (0.35% gains, ~ 0.1% losses) of the genome of normal tissue presents detectable CNAs. Arm-level (25%) and/or focal (10%) CNAs contribute to these observed aneuploidic changes. Together, these findings indicate that any locus is susceptible to change, especially in cancerous tissue, creating the potential for uncertainty in CNAs determined on the basis of a single reference locus.

We examined ways to minimize this uncertainty by employing a panel of reference loci as opposed to the pre-selection of a single reference. This strategy leverages the concept that although any given “reference” locus may exhibit a CNA within a clinical specimen, careful selection and analysis of a panel of loci whose CNA frequencies are, on average known to occur at or below the background frequency of aneuploidic CNAs in cancer (*i*.*e*. 33% [37]) should yield a subset of reference loci that remain stable during progression of a given cancer. For a panel of *N*_*tot*_ such candidate loci distributed across multiple chromosomes, S3 Table reports the probability (*p*_*stable*_) that at least *N*_*r*_ of those reference loci remain stable (CNA-neutral relative to the average ploidy of the tissue) during disease progression, assuming a 33% probability (*p*_*CNA*_) that any one of the reference loci will exhibit a CNA, where:

$$p_{stable}=\Sigma_{i=N_{r}}^{i=N_{tot}}\frac{N_{tot}!}{\left(N_{tot}-N_{r}\right)!}{\left(p_{CNA}\right)}^{N_{tot}-i}{\left(1-p_{CNA}\right)}^{i}$$ (9)

For a panel consisting of *N*_*tot*_ = 13 reference loci, as was used in the work reported here, more than half are predicted to remain stable in 90% of all samples, with at least *N*_*r*_ = 5 remaining stable in 99% of those samples. Creation of a truly stable benchmark for computing CNAs is therefore possible if one can establish a method for 1) identifying the subset of members of the panel of candidate reference loci that remain CNA-neutral and 2) averaging the data for the members of that subset to create a CNA-neutral benchmark.

We have created one such algorithm that has proven particularly reliable. To fix ideas, consider the generalized multi-well 4-plex ddPCR experiment where 4 templates are amplified in each well, and where one of those templates always is the constant reference *cr* (e.g., *HFE2*). The other targets analyzed in a given well are then selected from the set of reference loci, the length-based control series (e.g., *CPT2*) and the panel of chosen biomarkers. From the data collected for the length-based control, the set of ln(*R*_*i/cr*_)_*M*_ is computed using Eqs 1 and 2, and the required ln(*R*_*i/cr*_)_*M*_ versus Δ*l* plot constructed to allow each template to be corrected for statistically significant fragmentation (*m*_*frag*_ ≠ 0). Next, the ln(*R*_*i/cr*_)_*M*_ values for the *N*_*tot*_ candidate reference loci are computed in the same fashion, and then (if *m*_*frag*_ ≠ 0) corrected for fragmentation effects using Eqs 4 and 5 to obtain the mean *(R*_*i/cr*_)_*A*_ value and variance for each. While not essential, we find the dynamic range of statistically significant CNAs obtained by the method is increased by engineering all *N*_*tot*_ of the candidate reference panel to be of similar length—the 13 reference loci used here ranged from 92 to 113 bp in length.

**Fig 4:**
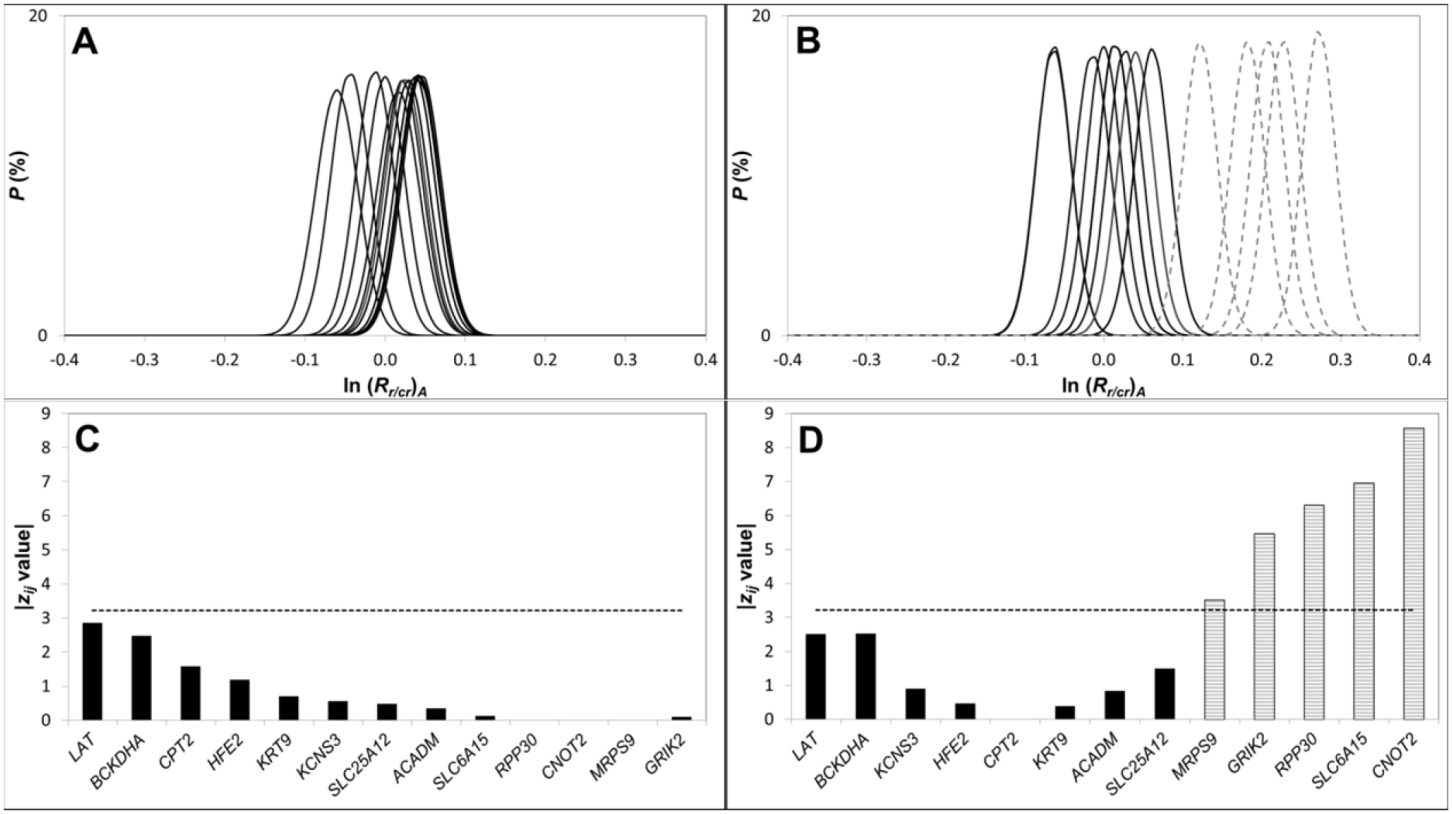
Selection of reference loci comprising a CNA-neutral benchmark. The ln (*R*_*i/cr*_)_*A*_ distributions for all 13 reference loci are reported for DNA extracted from (**A**) Area 4 (normal tissue) and (**B**) Area 2 (tissue displaying moderate to severe dysplasia (D3)). (**C**) The centroid reference locus *i* (*RPP30*) for the Area 4 sample and the |*z*_*ij*_| values comparing the centroid locus to each other reference loci. For this normal tissue specimen, the ln (*R*_*j/cr*_)_*A*_ for all reference loci *j* (≠ *i*) were statistically indistinguishable from that of the centroid locus *i* (all |*z*_*ij*_| < *z*_*c*_). (**D**) The same analysis applied to the Area 2 specimen, for which *CPT2* was determined to be the centroid locus and the ln (*R*_*j/cr*_)_*A*_ for a subset of 7 reference loci were found to be statistically indistinguishable from that of *CPT2*. For each sample *z*_*c*_ was computed assuming α = 0.05 and then correcting for multiple comparisons using the Bonferroni method.

For gDNA from frozen non-diseased connective tissue obtained in a field study of an OSCC patient, Fig 4A reports the *(R*_*i/cr*_)_*A*_ distributions for the *N*_*tot*_ candidate reference loci. As expected for this normal connective tissue, the mean *(R*_*i/cr*_)_*A*_ for each is near unity, but minor differences are observed.

The subset of these reference loci that are CNA-neutral may be defined through further analysis of their ln(*R*_*i/cr*_)_*A*_ distributions. That analysis begins by rank-ordering the set of candidate reference loci from lowest to highest ln(*R*_*i/cr*_)_*A*_ value. The subset *N*_*b*_ of loci that collectively best represents the average ploidy of the sample may then be identified by determining the locus *i* that is centroid of that subset, which is found by subjecting the set of ln(*R*_*i/cr*_)_*A*_ values to a k-means type clustering using one dimensional Euclidean distances. For rank-ordered reference *i* = 4, one computes the value of $\Delta \text{ln}{\left(R\right)}_{A_{i}}$ as

$$\Delta \text{ln}{\left(R\right)}_{A_{i}}=\Sigma_{j=1}^{3}\left(\text{ln}{\left(R_{\left(i+j\right)/cr}\right)}_{A}-\text{ln}{\left(R_{\left(i-j\right)/cr}\right)}_{A}\right)$$ (10)

This process is repeated for *i* = 5 to *i* = *N*_*tot*_− 3, and the centroid locus *i* then identified as that having the lowest $\Delta \text{ln}{\left(R\right)}_{A_{i}}$ value. A set of two-sample *z*-test values (*z*_*ij*_) comparing centroid locus *i* to each of the *j* = *N*_*tot*_− 1 other references is then computed as

$$z_{ij}=\frac{\left|\text{ln }{\left(R_{j/cr}\right)}_{A}-\text{ln }{\left(R_{i/cr}\right)}_{A}\right|}{\sqrt{{\left(\text{ln}{\left(\sigma_{R_{j/cr}}\right)}_{A}\right)}^{2}+{\left(\text{ln}{\left(\sigma_{R_{i/cr}}\right)}_{A}\right)}^{2}}}$$ (11)

where *j ≠ i*. It is a one-tailed test since the CNAs for the set of candidate reference loci have been rank-ordered.

The critical z value (*z*_*crit*_) for the resulting set of multiple sequential comparisons (78 in this example as the values have been rank ordered) is then computed through application of the Bonferroni method [46]. If, for example, we wish to identify the subset of reference loci that remain stable in 95% of all samples, an overall statistical significance (*α*) of 0.05 is applied. The Bonferroni method may then be used to define the adjusted significance (*α*_*Bon*_ = 0.05/78 = 0.000641) when comparing ln(*R)*_*A*_ values for each pair of candidate reference loci so as to maintain the required overall family-wise error rate. From this *α*_*Bon*_, *z*_*crit*_ (= 3.22 in this example) for a comparison of means is computed using established methods.

In Fig 4C, all candidate reference loci have a *z*_*ij*_ value below *z*_*crit*_, indicating that their ln(*R*_*j/cr*_)_*A*_ values are statistically indistinguishable from that of the centroid locus *i*. Thus, for this non-diseased connective tissue sample, all of the *N*_*tot*_ candidate reference loci are stable and may be collectively used to represent the average ploidy of the sample.

When we used the same analysis for a diseased tissue classified as moderate to severe dysplasia that was collected in a cancerous field of the same patient (Fig 4B), more pronounced differences in the ln(*R*_*i/cr*_)_*A*_ values for the set of candidate reference were observed. As a result, only a subset of the *N*_*tot*_ candidate reference loci then have a *z*_*ij*_ value below *z*_*crit*_ (Fig 4D), presumably due to CNAs in some of these loci as a consequence of global genetic instability associated with disease progression [47, 48].

This subset of *N*_*b*_ reference loci may be used to create a CNA-neutral benchmark *b*, where

$$\text{ln}{\left(R_{b/cr}\right)}_{A}=\frac{\sum_{i=1}^{N_{ss}}\text{ln}{\left(R_{i/cr}\right)}_{A}}{N_{ss}}$$ (12)

and

$$\text{ln}{\left(\sigma_{R_{b/cr}}\right)}_{A}=\sqrt{\frac{\sum_{i=1}^{N_{ss}}{\left[\text{ln}{\left(\sigma_{R_{i/cr}}\right)}_{A}\right]}^{2}}{N_{ss}}+\frac{\sum_{i=1}^{N_{ss}}{\left(\text{ln}{\left(R_{i/cr}\right)}_{A}-\text{ln}{\left(R_{b/cr}\right)}_{A}\right)}^{2}}{N_{ss}-1}}$$ (13)

Here, *i* is the index of the candidate reference loci within the subset.

With the benchmark defined, the copy number ratio *(R*_*i/b*_)_*A*_ for any biomarker *i* of interest may then be computed as

$$\text{ln}{\left(R_{i/b}\right)}_{A}=\text{ln}\left(\frac{{\left(R_{i/cr}\right)}_{A}}{{\left(R_{b/cr}\right)}_{A}}\right)$$ (14)

and the associated error as

$$\text{ln}{\left(\sigma_{R_{i/b}}\right)}_{A}=\sqrt{{\left(\text{ln}{\left(\sigma_{R_{i/cr}}\right)}_{A}\right)}^{2}+{\left(\text{ln}{\left(\sigma_{R_{b/cr}}\right)}_{A}\right)}^{2}}$$ (15)

Significant CNAs for each biomarker *i* can be determined using the null hypothesis for a set confidence interval (i.e. CI = 95%) and a two-sided tail analysis as the nature (gain or loss) of the CNA is now unknown.

### Application of Algorithm to Determination of CNAs in Frozen and FFPE Tissues, and Comparison with CGH Array Data

To verify our multiplexed ddPCR experimental design and data analysis methodology, we applied it to the determination of CNAs in biomarkers within DNA extracted from paired frozen and FFPE tissue specimens matched to samples analyzed previously by CGH array. *(R*_*i/b*_)_*A*_ values determined for each sample by ddPCR were normalized as (*R*_*i/b*_)_*A*_ − 1 for direct comparison to the corresponding log_2_ values determined by CGH array.

We first analyzed DNA from frozen tissue specimens collected as part of a larger study investigating somatic genomic alterations associated with oral cancer progression [38]. The set analyzed here is comprised of tissue collected at four histologically or geographically distinct areas within a cancerous field of one OSCC patient: Area 1 (SCC), Area 2 (moderate to severe dysplasia), Area 3 (SCC), and Area 4 (no dysplasia (normal) epithelial tissue). For three of those samples (Area 2, 3 and 4), DNA from the original extraction used in the CGH array study was available for the ddPCR analysis, while for the sample from Area 1, the required DNA was obtained from archived frozen tissue. Interestingly, very small but statistically significant levels of fragmentation (*m*_*frag*_ ≠ 0) were detected in the DNA previously extracted from biopsies of Areas 2, 3 and 4, while no statistically significant fragmentation (*m*_*frag*_ = 0) was observed in the freshly extracted DNA from Area 1. This suggests that long-term storage of DNA may contribute to minor fragmentation.

**Fig 5:**
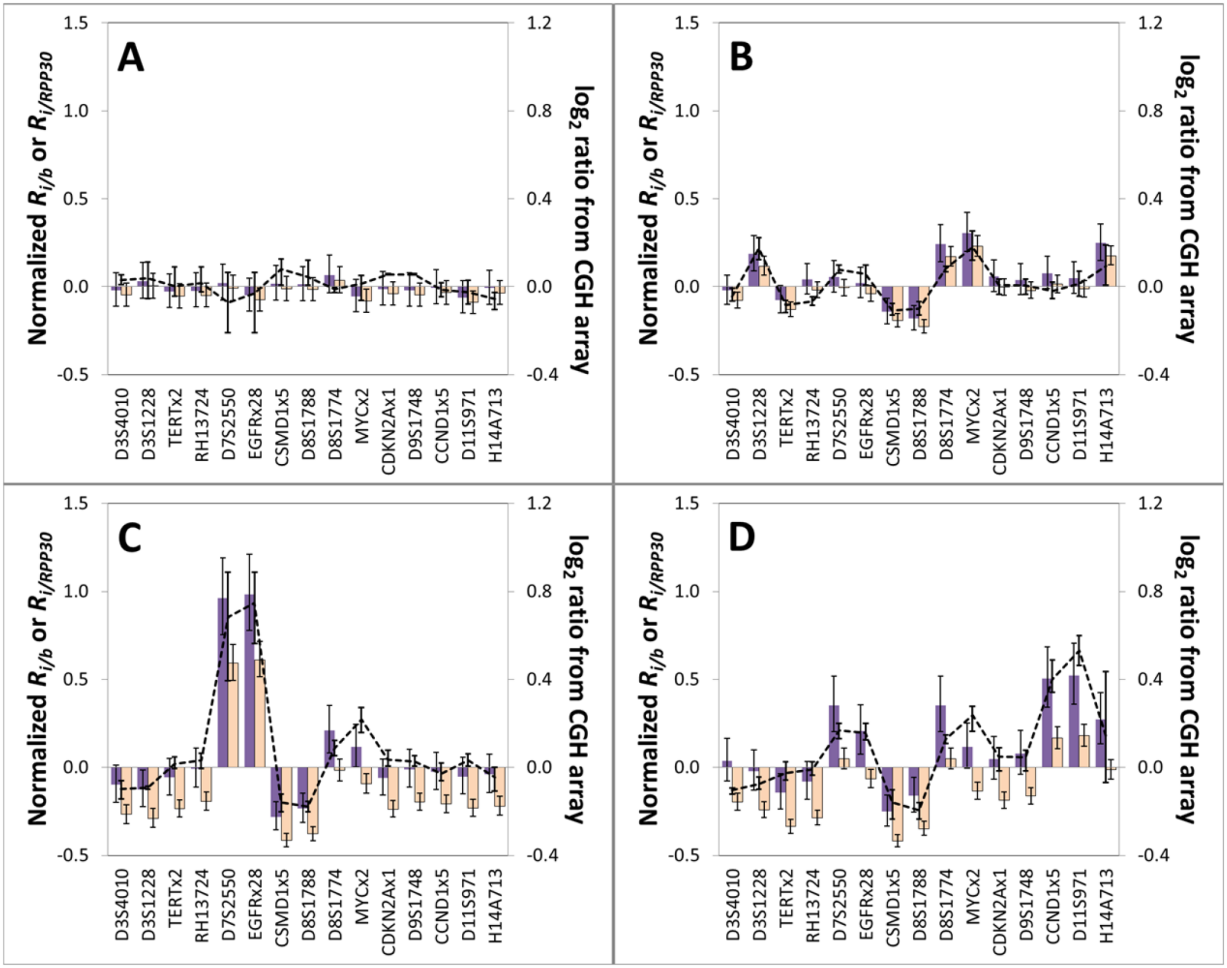
Comparison of CNAs measured within frozen tissue specimens by our multiplexed ddPCR method and by aCGH. 15 biomarkers within DNA extracted from four frozen tissue biopsies collected in a field study of the oral cavity of an oral cancer patient: **A** = Area 4 (normal connective tissue); **B** = Area 1 (OSCC-positive tissue); **C** = Area 2 (moderate to severe dysplasia); and **D** = Area 3 (OSCC-positive tissue). The log_2_ ratios from aCGH are the averaged values for the three or four probes that map closest to the biomarker interrogated by ddPCR (see Materials and Methods), with the error bars showing the respective high and low log_2_ ratios for these probes. Purple-filled bars are *R*_*i/b*_—1 (normalized) values computed using our CNA-neutral benchmark as reference and orange-filled bars are *R*_*i/RPP30*_*−*1 values computed using *RPP30* as a single reference locus. Error bars represent a 95% confidence interval. Biomarkers have been sorted by chromosomal location (x-axis).

For the normal (Area 4) tissue specimen, all of the 13 reference loci had statistically indistinguishable *(R*_*i/cr*_)_*A*_ values and were therefore used to define the CNA-neutral benchmark *(R*_*b/cr*_)_*A*_ (Fig 4A). In contrast, only a subset of the reference loci remains stable in the DNA from Areas 1, 2 and 3 (Fig 4 and S4 Fig). *RPP30*, which has often been used as a single reference locus for determining CNAs from digital PCR data [17, 22, 26], does not remain stable in all tissue samples. Rather, for the frozen tissue specimens from Areas 2 and 3, a statistically significant (*p* < 0.001) copy number gain in *RPP30* is recorded relative to the CNA-neutral benchmark (i.e., *R*_*RPP30/b*_− 1 > 0). This is problematic, as CNA determination by digital PCR is traditionally based on the pre-selection of a reference locus, such as *RPP30*. The undesirable impact this can have is illustrated by comparing normalized *R*_*i/b*_ values determined using either *RPP30* alone as the reference, versus using the CNA-neutral benchmark created from the panel of 13 reference loci (Fig 4 and S4 Fig). For all four frozen-tissue biopsy samples, normalized *R*_*i/b*_ values for each biomarker *i* show good agreement with the corresponding log_2_ values obtained from CGH array data when the CNA-neutral benchmark is applied (Fig 5 and S5 Fig). This does not hold true when *RPP30* alone is used as the reference locus. The normalized *R*_*i/b*_ (*b* = *RPP30*) values for the frozen tissue specimens from Areas 2 and 3 (Fig 5C and 5D, respectively) then deviate significantly from the corresponding log_2_ values.

**Fig 6:**
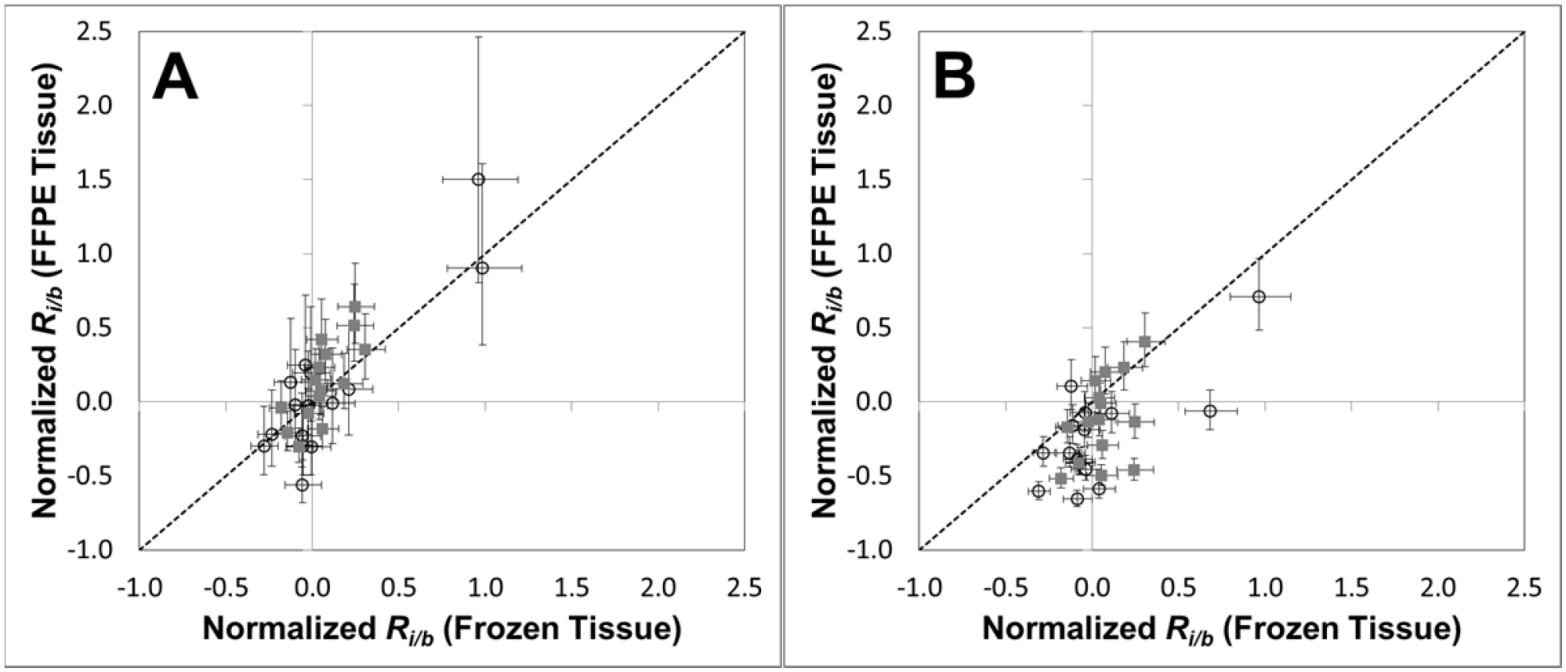
Comparison of CNAs in the same set of biomarkers in matched frozen and FFPE tissue specimens measured by our multiplexed ddPCR method. Mean normalized *R*_*i/b*_ values were determined for 15 biomarkers in DNA extracted either frozen or FFPE tissue biopsies of SCC in Area 1 (■) or the dysplasia in Area 2 (○). In general good agreement of normalized *R*_*i/b*_ values is observed between the matched samples when the fragmentation correction is applied (**A**), while a much poorer agreement is observed when the fragmentation correction is not applied (**B**). Error bars represent a 95% confidence interval.

Next, FFPE tissue biopsies from Areas 1 (SCC) and 2 (moderate to severe dysplasia) were used to determine if our method is also applicable to determination of CNAs within FFPE samples. In general, for each matched frozen and FFPE tissue sample, there is good agreement between the *R*_*i/b*_ values of the 15 biomarkers analyzed (Fig 6A). That agreement is observed despite the limited amount of DNA recovered from each FFPE specimen, and the highly fragmented state of that DNA (see S6 Fig). For Area 1, statistically significant CNAs observed in both the frozen and FFPE tissue include loss at 8p23.2 and gains at 8q24.21 (*MYC*) and 14q32.32, and for Area 2 loss at 8p23.2 and gain at *EGFR* (7p11.2); all of these CNAs also observed in the corresponding CGH array data. Finally, poor agreement of *R*_*i/b*_ values for the FFPE specimens with those for their matched frozen tissue is observed when the fragmentation correction is not applied (Fig 6B). As a result, the CGH-array confirmed gains in 8q24.21 and 14q32.32 are then not observed in the ddPCR results for the Area 1 FFPE specimen, illustrating the value of the fragmentation correction step in our method.

Although good agreement in CNA patterns obtained by our ddPCR method is observed between matched frozen and FFPE samples, some differences were noted, including variations in the reference loci selected for benchmark determination (S7 Fig) and certain statistically significant CNAs that were detected only in the FFPE specimens. For example in FFPE tissue from both Area 1 and 2, a significant loss was observed in the *TERTx2* (5p15.33) biomarker. As previously demonstrate by CGH arrays and other orthogonal analyses, CNAs in a patient’s oral cancer field are dynamic, with differences in CNA patterns often observed in biopsies from proximal areas within the same a cancerous field [38, 49]. Thus the slight differences in CNA patterns we observe are expected as a result of the intrinsic heterogeneity that exists within a cancerous field.

## Conclusions

While primarily used as a fundamental research tool, ddPCR, through its ability to quantify concentrations of amplifiable targets with high accuracy and sensitivity, is well suited for use in clinical assays. Indeed, ddPCR is finding ever-increasing use in assays to detect certain classes of somatic genomic mutations, most notably point mutations [50, 51] and translocations [44, 52] that are prognostic or theranostic of disease.

CNAs also correlate with disease, particularly cancers, but their accurate detection by ddPCR presents a unique and considerably greater challenge. It requires the quantification of (often subtle) changes in the abundances of germline loci thru an observed change in the corresponding abundances of loci-specific biomarkers relative to the average ploidy of the tissue. If that tissue section is formalin-fixed paraffin-embedded and sized such that only a limited amount of DNA can be extracted, as is often the case with clinical samples, application of ddPCR to CNA determination becomes even more difficult, due in part to the low quantity and quality of the DNA generally obtained. But it is possible, and the costs and throughput of dPCR are clinically attractive.

To advance ddPCR towards clinical application, we have reported on a strategy for designing multiplexed ddPCR assays that accurately quantify CNAs in a panel of prognostic biomarkers by minimizing experimental artifacts that can bias results. Determination of CNAs using this novel strategy requires method-specific data analysis, and we have established and then verified that analysis tool by demonstrating that CNAs computed with it are in agreement with data for the same samples acquired using established orthogonal methods (e.g. CGH arrays). The method, which corrects for gDNA fragmentation and also automatically selects from a panel of reference loci to establish a CNA-neutral benchmark, was successfully applied to both frozen and FFPE tissue specimens, demonstrating its ability to accurately detect CNAs in true clinical samples, which has not been demonstrated before.

## Supporting Information

S1 Fig The ddPCR output for monoplex amplification of the microsatellite biomarker *D4S1652* within DNA extracted from normal blood using either (A) standard primers or (B) primers optimized for multiplexed reactions. Although secondary rain is observed when standard primers are used in duplex reactions (see Fig 1), it is insignificant in standard monoplex ddPCR reactions.
(PDF)

S2 Fig An example of primary rain. The ddPCR output for the duplexed amplification of the reference locus *CPT2* (179 bp) (HEX) and the microsatellite biomarker locus *D3S3560* (probe 5’-FAM-aca+Caca+Cacaca+Cac-BHQ1-3’, and common RP 5’- tgcagttatgtatgagaacatcct-3’). Amplification of *D3S3560* used either (**A**) the FP 5’-ccttatgccctttgtcaaga-3’ or (**B**) the FP 5’-ccttatgccctttgccaaga-3’. A single nucleotide polymorphism aligns with the 3’ end (at the 3’-5 position) of the FP sequence, which results in a mismatch, reducing the efficiency of the PCR amplification of *D3S3560* to create primary rain. LNA bases are identified by capital letters preceded with plus (+) symbol.
(PDF)

S3 Fig An example of late rain. The ddPCR output for the duplexed amplification of the reference locus *CPT2* (179 bp) (HEX) and the microsatellite biomarker locus *RH808* (FP 5’-aaatcactcctgcttgatctc-3’ and RP 5’- gggcagactccctctagtaa-3’). Amplification of *RH808* was detected with either (**A**) a 10-mer LNA substituted probe 5’-FAM-a+Ca+C+A+Ca+C+Ac-BHQ1-3’ or (**B**) a 16-mer LNA substituted probe 5’-FAM-aca+Caca+Cacaca+Cac-BHQ1-3’. The short length of the 10-mer LNA substituted probe results in late rain due non-specific hybridization and hydrolysis. LNA bases are identified by capital letters preceded with plus (+) symbol.
(PDF)

S4 Fig Selection of reference loci comprising a CNA-neutral benchmark for DNA from frozen tissue specimens. The ln(*R*_*i/cr*_)_*A*_ distributions for all 13 reference loci are reported for DNA extracted from frozen tissue from (**A**) Area 1 (SCC) or (**B**) Area 3 (SCC). (**C**) The centroid reference locus *i* (*CPT2*) for the Area 1 sample and the |*z*_*ij*_| values comparing the centroid locus to each other reference loci. For this sample the ln(*R*_*j/cr*_)_*A*_ for 9 of the reference loci *j* (≠ *i*) were statistically indistinguishable from that of the centroid locus *i* (|*z*_*ij*_| < *z*_*c*_). (**D**) The centroid reference locus *i* (*KCNS3*) for the Area 3 sample and the |*z*_*ij*_| values comparing the centroid locus to each other reference loci. For this sample the ln(*R*_*j/cr*_)_*A*_ for 6 of the reference loci *j* (≠ *i*) were statistically indistinguishable from that of the centroid locus *i* (|*z*_*ij*_| < *z*_*c*_). For each sample *z*_*c*_ was computed assuming α = 0.05 and then correcting for multiple comparisons using the Bonferroni method.
(PDF)

S5 Fig Comparison of log_2_ ratio from aCGH to the corresponding normalized *R*_*i/b*_ values from analysis of ddPCR. Results for DNA extracted from frozen tissue taken from ▲Area 1 (SCC); ○ Area 2 (D3); ● Area 3 (SCC) and ■ Area 4 (Normal). Analysis of the trend line for all data points yields the following linear relationship: log_2_ = 0.7 *R*_*i/b*_ + 0.0, which was used to calibrate the y-axis scales in Fig 6. The log_2_ ratios are the averaged values for the 3 or 4 probes that map closest to the biomarker interrogated by ddPCR (see Materials and Methods). Error bars are the high and low log_2_ ratios for these probes. Horizontal error bars represent a 95% confidence interval in the *R*_*i/b*_ values.
(PDF)

S6 Fig Analysis and comparison of fragmentation in DNA taken from Area 1 and 2 for frozen and FFPE tissue specimens. The ln(*R*_*CPT2(l)/HFE2*_) values from ddPCR experiments on gDNA recovered from frozen (open symbols) or FFPE (filled symbols) tissue specimens for Area 1 (circles) and Area 2 (triangles). Error bars represent a 95% confidence interval.
(PDF)

S7 Fig Selection of reference loci comprising a CNA-neutral benchmark for FFPE tissue specimens. The ln(*R*_*i/cr*_)_*A*_ distributions for all 13 reference loci are reported for DNA extracted from FFPE tissue biopsies from (**A**) Area 1 (SCC) and (**B**) Area 2 (moderate to severe dysplasia). (**C**) The centroid reference locus *i* (*KCNS3*) for the Area 1 sample and the |*z*_*ij*_| values comparing the centroid locus to each other reference loci. For this sample the ln(*R*_*j/cr*_)_*A*_ for 9 of the reference loci *j* (≠ *i*) were statistically indistinguishable from that of the centroid locus *i* (|*z*_*ij*_| < *z*_*c*_). (**D**) The centroid reference locus *i* (*RPP30*) for the Area 2 sample and the |*z*_*ij*_| values comparing the centroid locus to each other reference loci. For this sample the ln(*R*_*j/cr*_)_*A*_ for 10 of the reference loci *j* (≠ *i*) were statistically indistinguishable from that of the centroid locus *i* (|*z*_*ij*_| < *z*_*c*_). For each sample *z*_*c*_ was computed assuming α = 0.05 and then correcting for multiple comparisons using the Bonferroni method.
(PDF)

S1 Table Primers and probes used in ddPCR experiments. (PDF)

S2 Table Identification and explanation of the types of rain associated with suboptimal reactions observed in the output of ddPCR experiments. (PDF)

S3 Table Setting the size of the reference loci panel. Minimum number of stable reference loci (*N*_*r*_) predicted by eq 9 for different values of *p*_*stable*_ assuming *p*_*CNA*_ = 33% and as a function of the total number of reference loci (*N*_*tot*_) used.
(PDF)
