# Supplementary figures and images for: A Robust Protocol for Using Multiplexed Droplet Digital PCR to Quantify Somatic Copy Number Alterations in Clinical Tissue Specimens
Source: PLoS One. 2016 Aug 18;11(8):e0161274. doi: 10.1371/journal.pone.0161274 (PMC4990255; doi:10.1371/journal.pone.0161274)

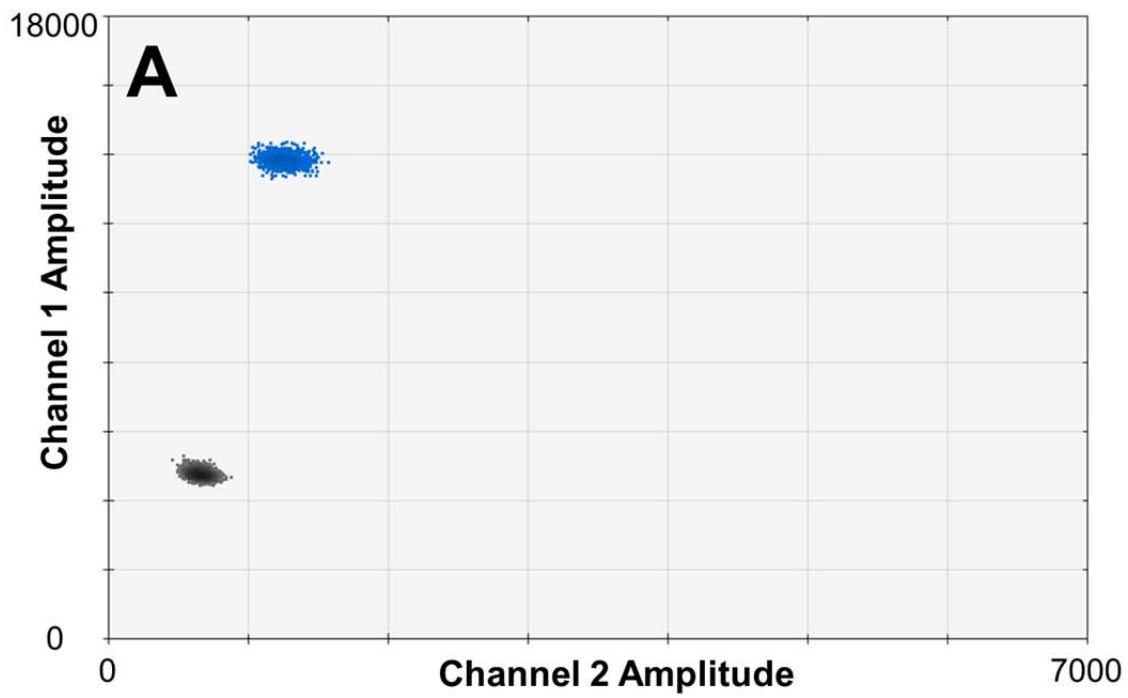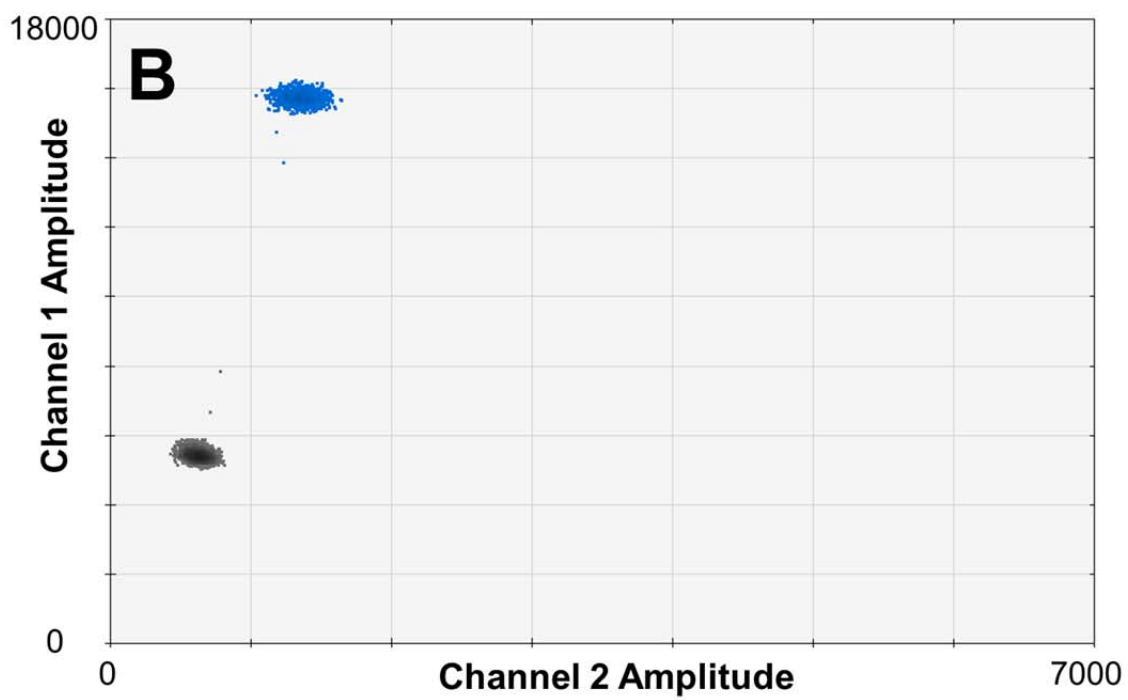

Supplement: S1 Fig — Although secondary rain is observed when standard primers are used in duplex reactions (see Fig 1), it is insignificant in standard monoplex ddPCR reactions. (PDF) [file pone.0161274.s001.pdf]

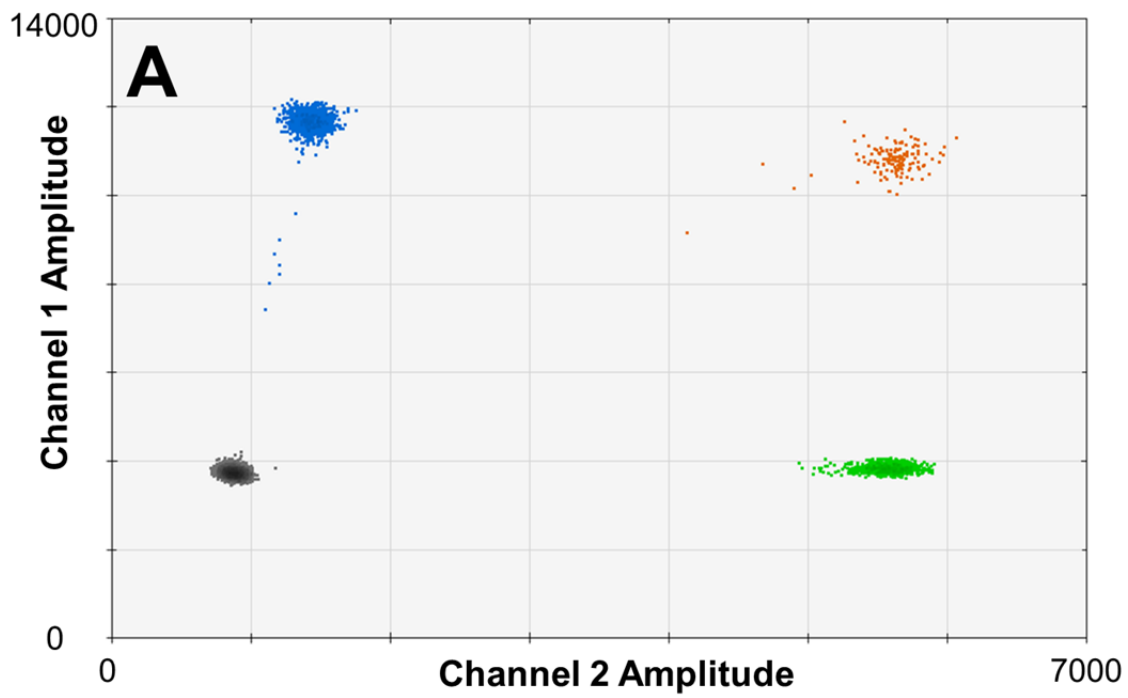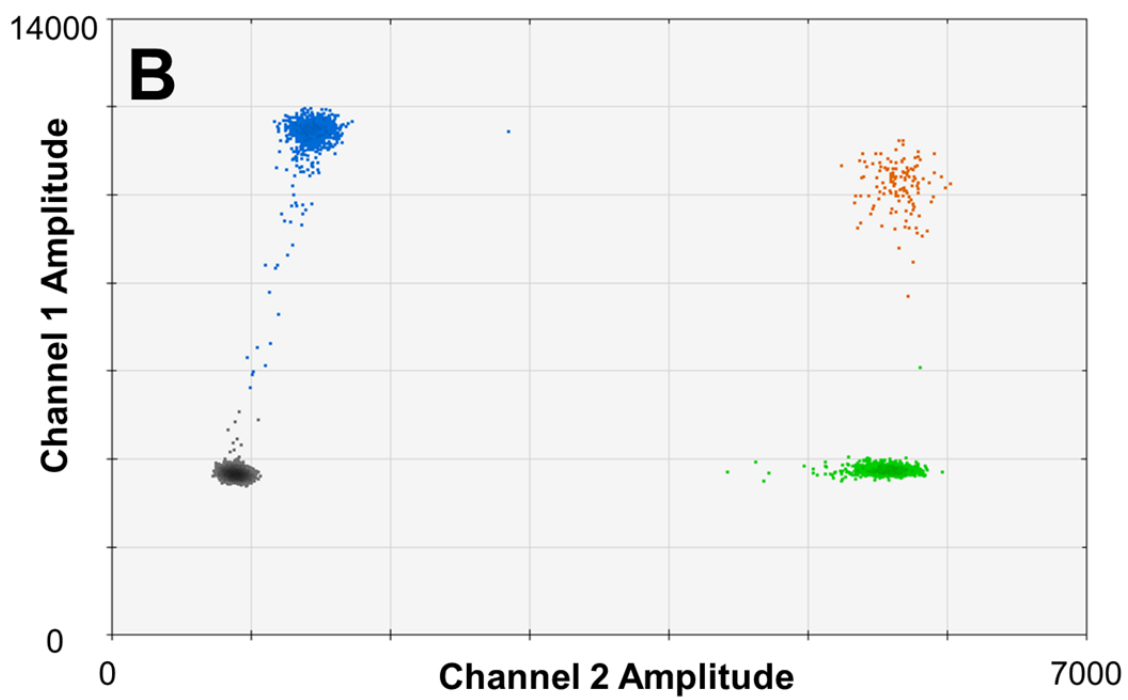

Supplement: S2 Fig — The ddPCR output for the duplexed amplification of the reference locus CPT2 (179 bp) (HEX) and the microsatellite biomarker locus D3S3560 (probe 5’-FAM-aca+Caca+Cacaca+Cac-BHQ1-3’, and common RP 5’- tgcagttatgtatgagaacatcct-3’). Amplification of D3S3560 used either (A) the FP 5’-ccttatgccctttgtcaaga-3’ or (B) the FP 5’-ccttatgccctttgccaaga-3’. A single nucleotide polymorphism aligns with the 3’ end (at the 3’-5 position) of the FP sequence, which results in a mismatch, reducing the efficiency of the PCR amplification of D3S3560 to create primary rain. LNA bases are identified by capital letters preceded with plus (+) symbol. (PDF) [file pone.0161274.s002.pdf]

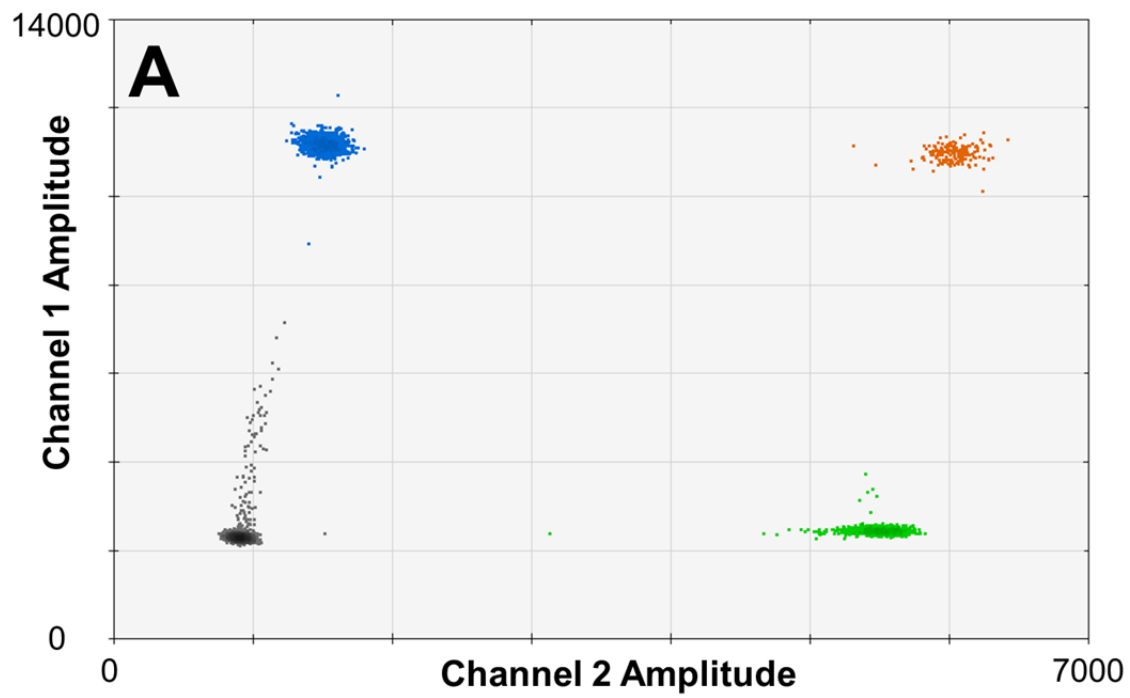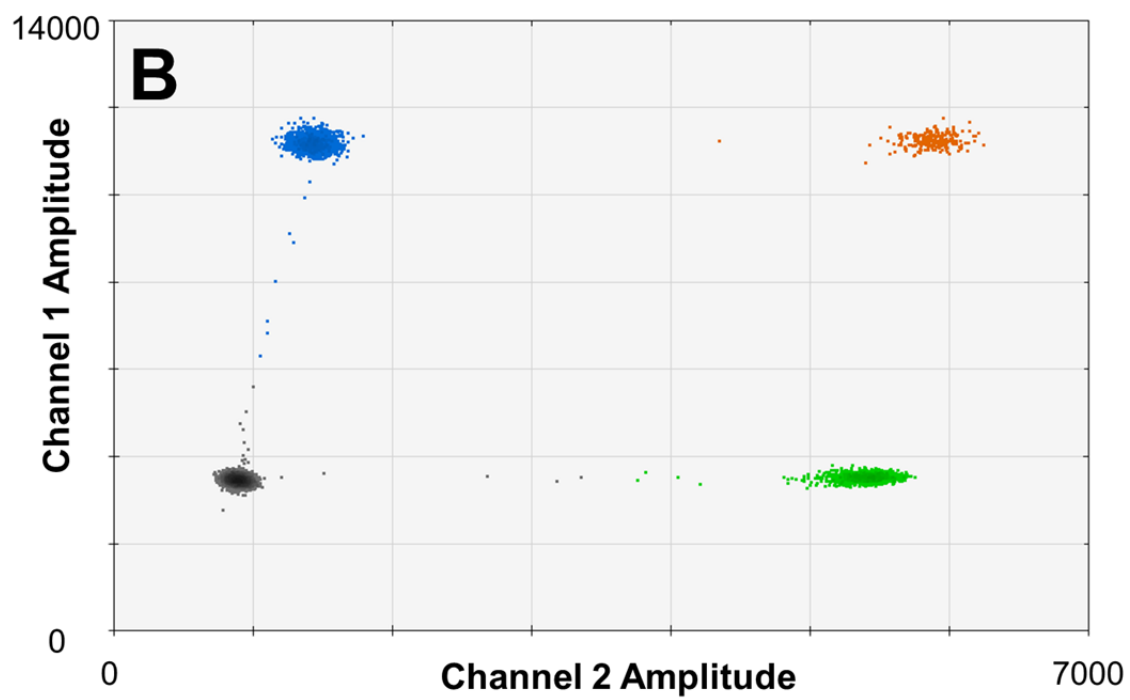

Supplement: S3 Fig — The ddPCR output for the duplexed amplification of the reference locus CPT2 (179 bp) (HEX) and the microsatellite biomarker locus RH808 (FP 5’-aaatcactcctgcttgatctc-3’ and RP 5’- gggcagactccctctagtaa-3’). Amplification of RH808 was detected with either (A) a 10-mer LNA substituted probe 5’-FAM-a+Ca+C+A+Ca+C+Ac-BHQ1-3’ or (B) a 16-mer LNA substituted probe 5’-FAM-aca+Caca+Cacaca+Cac-BHQ1-3’. The short length of the 10-mer LNA substituted probe results in late rain due non-specific hybridization and hydrolysis. LNA bases are identified by capital letters preceded with plus (+) symbol. (PDF) [file pone.0161274.s003.pdf]

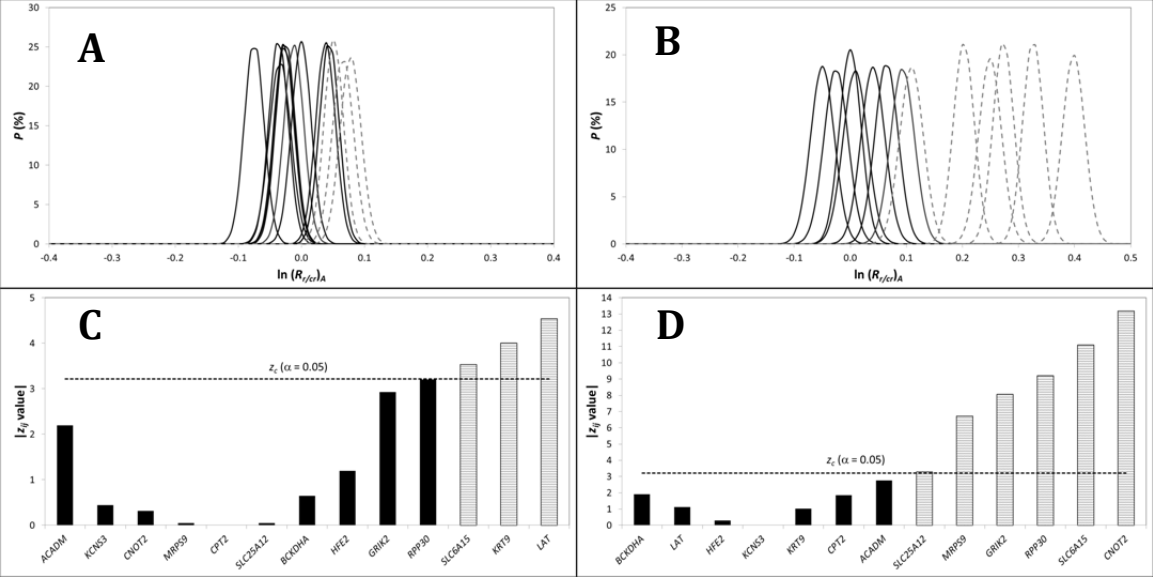

Supplement: S4 Fig — The ln(Ri/cr)A distributions for all 13 reference loci are reported for DNA extracted from frozen tissue from (A) Area 1 (SCC) or (B) Area 3 (SCC). (C) The centroid reference locus i (CPT2) for the Area 1 sample and the |zij| values comparing the centroid locus to each other reference loci. For this sample the ln(Rj/cr)A for 9 of the reference loci j (≠ i) were statistically indistinguishable from that of the centroid locus i (|zij| < zc). (D) The centroid reference locus i (KCNS3) for the Area 3 sample and the |zij| values comparing the centroid locus to each other reference loci. For this sample the ln(Rj/cr)A for 6 of the reference loci j (≠ i) were statistically indistinguishable from that of the centroid locus i (|zij| < zc). For each sample zc was computed assuming α = 0.05 and then correcting for multiple comparisons using the Bonferroni method. (PDF) [file pone.0161274.s004.pdf]

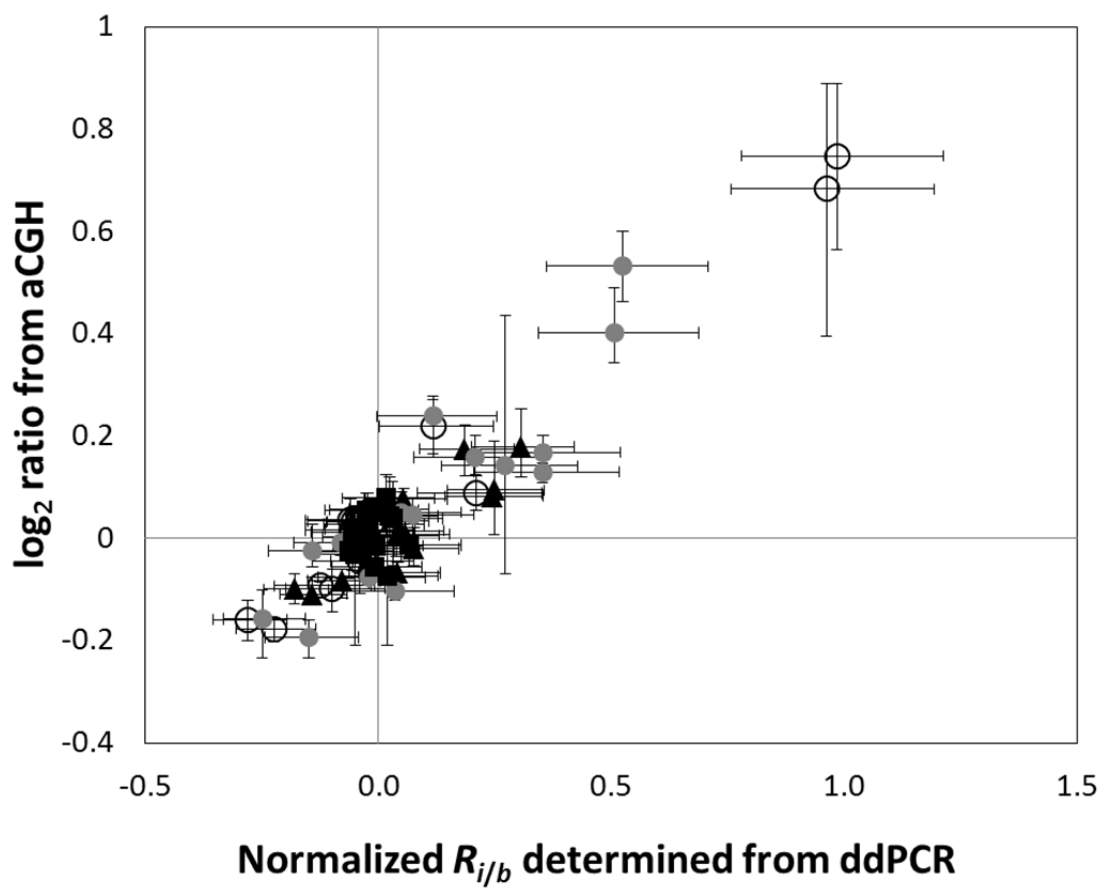

Supplement: S5 Fig — Results for DNA extracted from frozen tissue taken from ▲Area 1 (SCC); ○ Area 2 (D3); ● Area 3 (SCC) and ■ Area 4 (Normal). Analysis of the trend line for all data points yields the following linear relationship: log2 = 0.7 Ri/b + 0.0, which was used to calibrate the y-axis scales in Fig 6. The log2 ratios are the averaged values for the 3 or 4 probes that map closest to the biomarker interrogated by ddPCR (see Materials and Methods). Error bars are the high and low log2 ratios for these probes. Horizontal error bars represent a 95% confidence interval in the Ri/b values. (PDF) [file pone.0161274.s005.pdf]

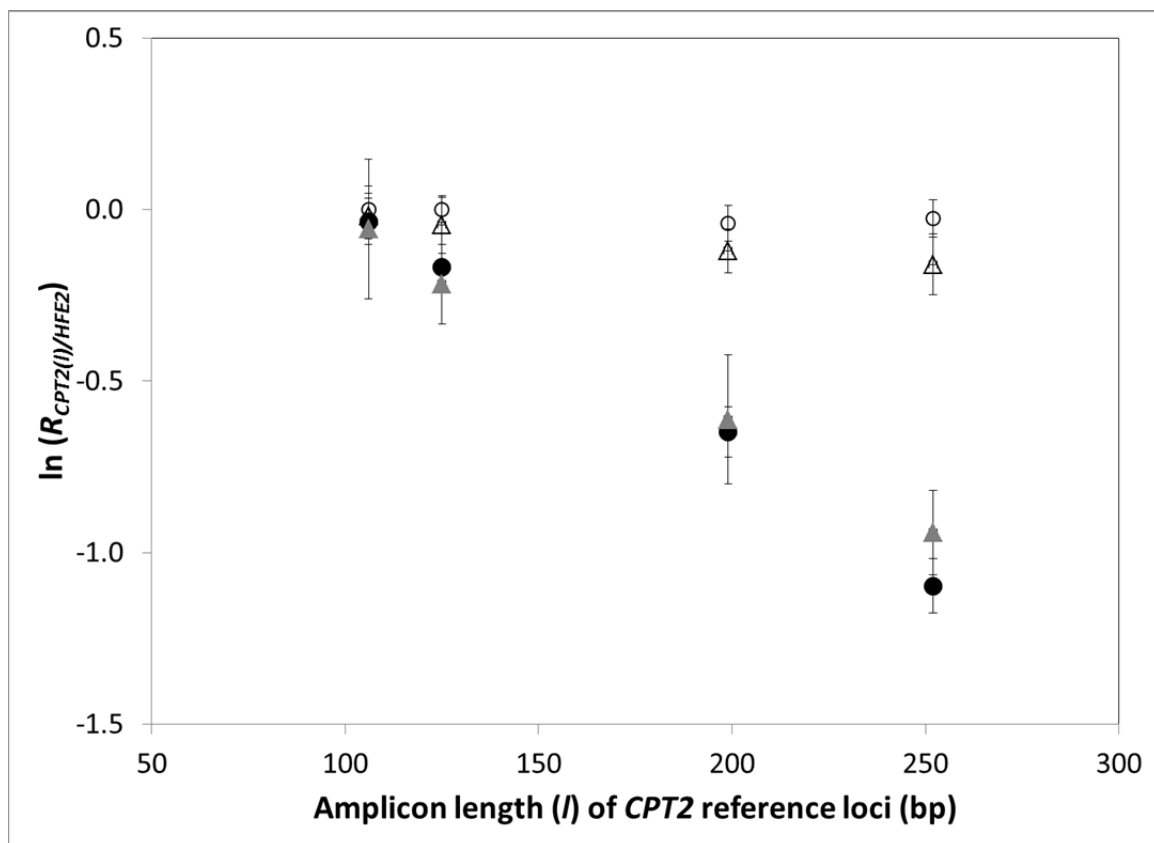

Supplement: S6 Fig — The ln(RCPT2(l)/HFE2) values from ddPCR experiments on gDNA recovered from frozen (open symbols) or FFPE (filled symbols) tissue specimens for Area 1 (circles) and Area 2 (triangles). Error bars represent a 95% confidence interval. (PDF) [file pone.0161274.s006.pdf]

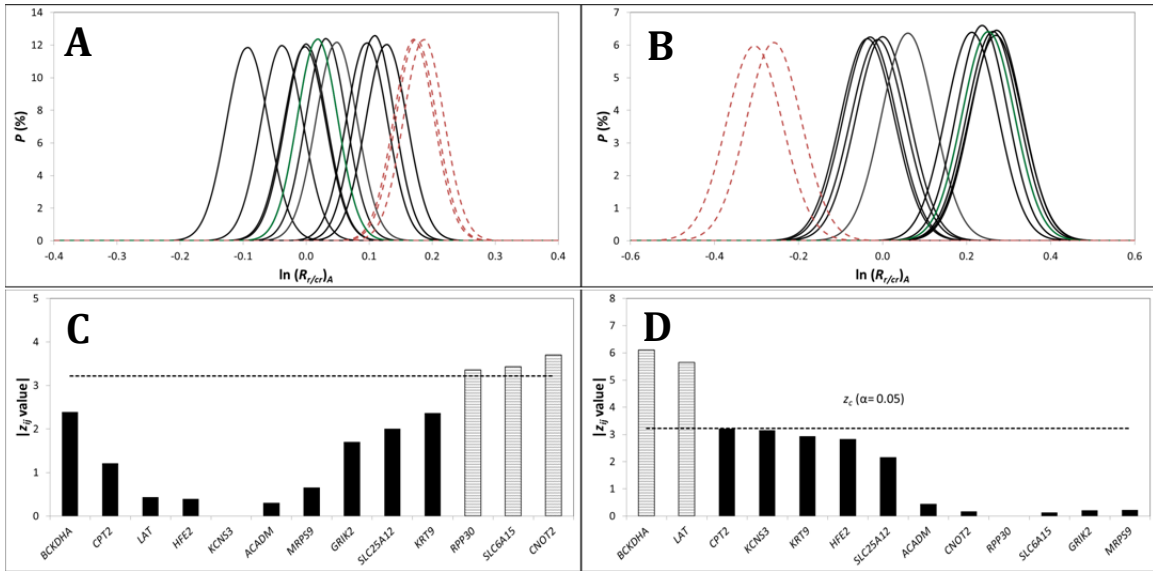

Supplement: S7 Fig — The ln(Ri/cr)A distributions for all 13 reference loci are reported for DNA extracted from FFPE tissue biopsies from (A) Area 1 (SCC) and (B) Area 2 (moderate to severe dysplasia). (C) The centroid reference locus i (KCNS3) for the Area 1 sample and the |zij| values comparing the centroid locus to each other reference loci. For this sample the ln(Rj/cr)A for 9 of the reference loci j (≠ i) were statistically indistinguishable from that of the centroid locus i (|zij| < zc). (D) The centroid reference locus i (RPP30) for the Area 2 sample and the |zij| values comparing the centroid locus to each other reference loci. For this sample the ln(Rj/cr)A for 10 of the reference loci j (≠ i) were statistically indistinguishable from that of the centroid locus i (|zij| < zc). For each sample zc was computed assuming α = 0.05 and then correcting for multiple comparisons using the Bonferroni method. (PDF) [file pone.0161274.s007.pdf]
