# Supplementary material for: A Robust Protocol for Using Multiplexed Droplet Digital PCR to Quantify Somatic Copy Number Alterations in Clinical Tissue Specimens
Source: PLoS One. 2016 Aug 18;11(8):e0161274. doi: 10.1371/journal.pone.0161274 (PMC4990255; doi:10.1371/journal.pone.0161274)
Supplement: S1 Table — (PDF) [file pone.0161274.s008.pdf]

Locus	l^1 (bp)	Forward Primer[2]	Reverse Primer[2]	Probe[2,3]
HFE2	97	gggatccagtttgtcgattca	agctgtctgccgaatgattatag	aactgctaaccctgggaaccatgt
CPT2	106	ccagcagtgaaccttggg	ctgggtaggaagagacattgc	cctgtggtctctgatggctttggt
	125	tacgggcagataaaccacaa	cagttgtcatgaacagcatacc	cctgtggtctctgatggctttggt
	151	cctgcatacgggcagataaa	aagagacattgcagcctatcc	cctgtggtctctgatggctttggt
	179	gatcatcttgcctgagctctac	aagagacattgcagcctatcc	cctgtggtctctgatggctttggt
	199	tacgggcagataaaccacaat	ctaaggccttctccacacatt	cctgtggtctctgatggctttggt
	252	tgaccgacacttgtttgctctg	aactcccgggcattgcg	cctgtggtctctgatggctttggt
KCNS3	101	gcttatcgctatctcctccttg	catccacttctccatcctcatt	cgttcacagcatgtcggagttcca
	124	tcctccttgagcgtggt	caatgcacgcgatctcca	cgttcacagcatgtcggagttcca
	154	gtactgcctgtccgctaag	aatgcacgcgatctcca	cgttcacagcatgtcggagttcca
	178	ggtcagctccggaagaaa	cttccagcaccggatcat	cgttcacagcatgtcggagttcca
	233	gtgctggcctccatcgt	acagccaacgtggcatagaa	cgttcacagcatgtcggagttcca
	278	ccagcgtactgcctgtc	aacgtggcatagaagggaataa	cgttcacagcatgtcggagttcca
ACADM	101	gatgttcagatactagaggaattgt	agctcccattgcaacttt	ttaattggtgacggagctggtttc
MRPS9	100	aacgtgaaacttacacagagga	gtttctggatcttctcccatca	ctctttcctatgttgaactcttcaatctgc
SLC25A12	106	gatcacatcggtggatacagac	gaaccacagcaacactagga	tttcgatgcctgcaaacgtggc
GRIK2	99	gacctctttgctcttgatgttg	gatggaggagacttgggtattt	aacaccactgtatcggtagggct
RPP30	98	gggaaggaagtatgacagatgtt	gaagccatccttgagtccttag	agcaaagtacaacaggaagacaccttgg
CNOT2	113	ggaatatgatgaaccactccca	tattatgcttggcgagcttct	agcatgagcagttcagggttaggt
SLC6A15	98	tgttcgtcgcttcaaccttat	cctctaagttcacaggctctttc	acccttcctctcttataggtcacagatgc
LAT	97	cgtcctggactaggctga	aggtagcctgggttgtgata	cccagaaccagcctgtgaggat
KRT9	92	gctggcctcttacttggataag	gtcccttcttgtcgtaccaatc	attctccaggtcgttgttggcctc
BCKDHA	105	caccagtgacgacagttcag	ccttggctcagcagatagtg	ccgctcggtggatgaggtcaatta
D4S1652 (Standard)	144	aatccctgggtacattatatttg	cagacattctttattctttacctcc	at+Ctat+Cta+T+Ctat+Ctat+Ct
D4S1652 (Optimized)	146	ggaatccctgggtacattatatttg	cagacattctttattctttacctcc	at+Ctat+Cta+T+Ctat+Ctat+Ct
D3S1234	111	cctgtgagacaaagcaagac	gacattaggcacagggctaa	a+Ca+Ca+Ca+Cac+Ac
D3S1300	247	agctcacattctagtcagcct	gaatgccaattccccagatg	a+Ca+Ca+Ca+Cac+Ac
D3S1300sh	128	agctcacattctagtcagcct	ccacatgacgttcctttgtagt	a+Ca+Ca+Ca+Cac+Ac
D3S4010	108	cacctccaccactgcaatc	tcccttgtttttgggaatctt	a+T+Catca+T+Catca+T+Catc
D3S1228	86	tccttaactctttctctgtgagttg	tctaggaaagggattaggaagga	a+Ca+Ca+Ca+Cac+Ac
TERTx2	124	gaccaagcacttcctctactc	ggaacccagaaagatggtctc	agagagctgagtaggaaggagggc
RH13724	119	tcagaatcatttctcaatagcc	actccaggagcatctcaaat	a+Ca+Ca+Ca+Cac+Ac
D7S2550	259	aagttccatttgtctcggtt	agtctcctcgtctcacacct	a+Ca+Ca+Ca+Cac+Ac
EGFRx28	100	cattagctcttagacccacagac	caaaggaatgcaacttcccaaa	tgcaacgtttacaccgactagcca
D8S1788	209	agttcaagcctagtttcataaaag	aagactcctaatgtctatttttcag	a+Ca+Ca+Ca+Cac+Ac
CSMD1x5	93	cccttcagagtacgagaacaac	agctgaaagtcagtgaagacc	cggactgcacctggaccattct
D8S1774	262	tgcataggttgctgactttg	cctgaggcttaggcattattag	a+Ca+Ca+Ca+Cac+Ac
MYCx2	94	gagacatggtgaaccagagtt	gagaagccgctccacatac	ccggacgacgagaccttcatcaaa
CDKN2Ax1	120	ggagccttcggctgact	atcggcctccgaccgtaa	tattcggtgcgttgggcagc
D9S1748	125	cacctcagaagtcagtgagt	gtgcttgaaatacacctttcc	a+Ca+Ca+Ca+Cac+Ac
D9S1751	178	ttgttgattctgccttcaaagtcttttaac	cgttaagtcctctattacacagag	a+Ca+Ca+Ca+Cac+Ac
D9S171	175	agctaagtgaacctcatctctgtct	accctagcactgatggtatagtct	a+Ca+Ca+Ca+Cac+Ac
CCND1x5	113	gaggatgttcataaggccagta	ctgtaacatcaaaggcagaagg	a+Ca+Ca+Ca+Cac+Ac
D11S971	111	aggacacagcctgcatctag	accaggcattgcactaaaag	ttcat+T+Cat+T+Cat+T+Cattca
H14A713	191	aacccaaaccacgagatacc	gtgccgacgtcataacaagg	a+Ca+Ca+Ca+Cac+Ac

[1] Amplicon lengths determined from primer blast (www.ncbi.nlm.nih.gov/tools/primer-blast/) and/or from the UCSC genome browser (https://genome.ucsc.edu/) For microsatellite biomarkers with a reported size range, the amplicon length used is the median of the range.

[2] Sequences reported in 5' to 3' direction. LNA bases are identified by capitals and a preceding + symbol.

[3] All DNA AND LNA probes were labelled with either 5' FAM or 5'-HEX. DNA probes contained an internal ZEN quencher and a 3'-IBFQ, while LNA probes contained only a 3'-quencher (IBFQ or BHQ1).
