# Supplementary material for: A Robust Protocol for Using Multiplexed Droplet Digital PCR to Quantify Somatic Copy Number Alterations in Clinical Tissue Specimens
Source: PLoS One. 2016 Aug 18;11(8):e0161274. doi: 10.1371/journal.pone.0161274 (PMC4990255; doi:10.1371/journal.pone.0161274)
Supplement: S2 Table — (PDF) [file pone.0161274.s009.pdf]

Rain Type	Description
Primary Rain	• ***Characterization:*** Directional rain affecting the primary cluster and associated higher order cluster(s)<br>• ***Origin:*** Inefficient PCR<br>• ***Modification:*** Redesign probe with higher T_m and/or check primer and probe hybridization sites for common SNPs and SPMs<br>• ***Observed:*** Both monoplex and multiplexed ddPCR<br>• ***Example:*** Figure S2
Secondary Rain	• ***Characterization:*** Directional rain affecting only higher order cluster(s)<br>• ***Origin:*** Significant differences in the PCR efficiency between two or more target and/or reference templates.<br>• ***Modification:*** Lengthen primer(s) to increase T_m<br>• ***Observed:*** Only in multiplexed ddPCR<br>• ***Example:*** Figure 1 and S1
Late Rain	• ***Characterization:*** Directional rain that appears to originate from the "empty" or no template droplet cluster<br>• ***Origin:*** Non-specific template amplification resulting in probe hydrolysis<br>• ***Modification:*** Alter probe length and/or sequence<br>• ***Observed:*** Generally in multiplexed ddPCR; possible in monoplex<br>• ***Example:*** Figure S3
