# Supplementary material for: A Robust Protocol for Using Multiplexed Droplet Digital PCR to Quantify Somatic Copy Number Alterations in Clinical Tissue Specimens
Source: PLoS One. 2016 Aug 18;11(8):e0161274. doi: 10.1371/journal.pone.0161274 (PMC4990255; doi:10.1371/journal.pone.0161274)
Supplement: S3 Table — Minimum number of stable reference loci (Nr) predicted by eq 9 for different values of pstable assuming pCNA = 33% and as a function of the total number of reference loci (Ntot) used. (PDF) [file pone.0161274.s010.pdf]

N_{tot}	Minimum number of stable reference loci (N_r) when p_{stable} = 90%	p_{stable} = 95%	p_{stable} = 99%
**4**	1	1	1
**7**	3	3	2
**10**	5	4	3
**13**	7	6	5
**16**	8	8	6
